# Towards Predicting Basin-Wide Invertebrate Organic Biomass and Production in Marine Sediments from a Coastal Sea

**DOI:** 10.1371/journal.pone.0040295

**Published:** 2012-07-06

**Authors:** Brenda J. Burd, Tara A. Macdonald, Albert van Roodselaar

**Affiliations:** 1 Institute of Ocean Sciences, Sidney, British Columbia, Canada; 2 Metro Vancouver, Kingsway Burnaby, British Columbia, Canada; Utrecht University, The Netherlands

## Abstract

Detailed knowledge of environmental conditions is required to understand faunal production in coastal seas with topographic and hydrographic complexity. We test the hypothesis that organic biomass and production of subtidal sediment invertebrates throughout the Strait of Georgia, west coast of Canada, can be predicted by depth, substrate type and organic flux modified to reflect lability and age of material. A basin-wide database of biological, geochemical and flux data was analysed using an empirical production/biomass (P/B) model to test this hypothesis. This analysis is unique in the spatial extent and detail of P/B and concurrent environmental measurements over a temperate coastal region. Modified organic flux was the most important predictor of organic biomass and production. Depth and substrate type were secondary modifiers. Between 69–74% of variability in biomass and production could be explained by the combined environmental factors. Organisms <1 mm were important contributors to biomass and production primarily in shallow, sandy sediments, where high P/B values were found despite low organic flux. Low biomass, production, and P/B values were found in the deep, northern basin and mainland fjords, which had silty sediments, low organic flux, low biomass of organisms <1 mm, and dominance by large, slow-growing macrofauna. In the highest organic flux and biomass areas near the Fraser River discharge, production did not increase beyond moderate flux levels. Although highly productive, this area had low P/B. Clearly, food input is insufficient to explain the complex patterns in faunal production revealed here. Additional environmental factors (depth, substrate type and unmeasured factors) are important modifiers of these patterns. Potential reasons for the above patterns are explored, along with a discussion of unmeasured factors possibly responsible for unexplained (30%) variance in biomass and production. We now have the tools for basin-wide first-order estimates of sediment invertebrate production.

## Introduction

Over the past 9 years, a collaborative research project between Metro Vancouver, Fisheries and Oceans Canada and Natural Resources Canada (see [1] and references therein) has focused on understanding and modeling carbon and contaminant cycling in the Strait of Georgia, an inland sea spanning most of the populated west coast of Canada. One of the project goals is to develop a baseline understanding of background biological function in sediments of the Strait, in order to provide context for assessing the extent and importance of anthropogenic inputs of organic carbon and contaminants, and to be able to detect fundamental changes related to shifting background conditions due to climate change. Detecting climate-related changes in functioning of marine sediments requires an understanding of present-day conditions and how these are affected by natural factors. [2].

As part of the collaborative project, Johannessen et al. [3] proposed a preliminary organic carbon budget for the Strait of Georgia. In that budget, the sediments were depicted as an important sink for organic carbon, with an unknown proportion of the measured organic carbon flux to the bottom held within benthic biota and/or returned to the water column through trophic exchange (c.f.[4],[5]), respiration and reproduction. To fill this gap, it is necessary to understand inventory (organic biomass) and cycling (production) of organic material in sediments, and how this is influenced by present-day environmental conditions.

Although benthic species assemblages may shift considerably over space and time, the degree to which benthic biomass and production are conservative (predictable) across broad geographic regions relative to basic geo-morphological features such as depth, sediment type, food input and quality, will determine how adaptable and healthy benthic assemblages in coastal regions are likely to remain under changing conditions. These environmental factors tend to encompass or integrate the effects of many other factors (bottom currents, sediment oxygenation, storms, light penetration, exposure, physical stability, etc.), and are thus expected to critical for structuring patterns in marine sediment biomass and production on a regional scale.

**Figure 1:**
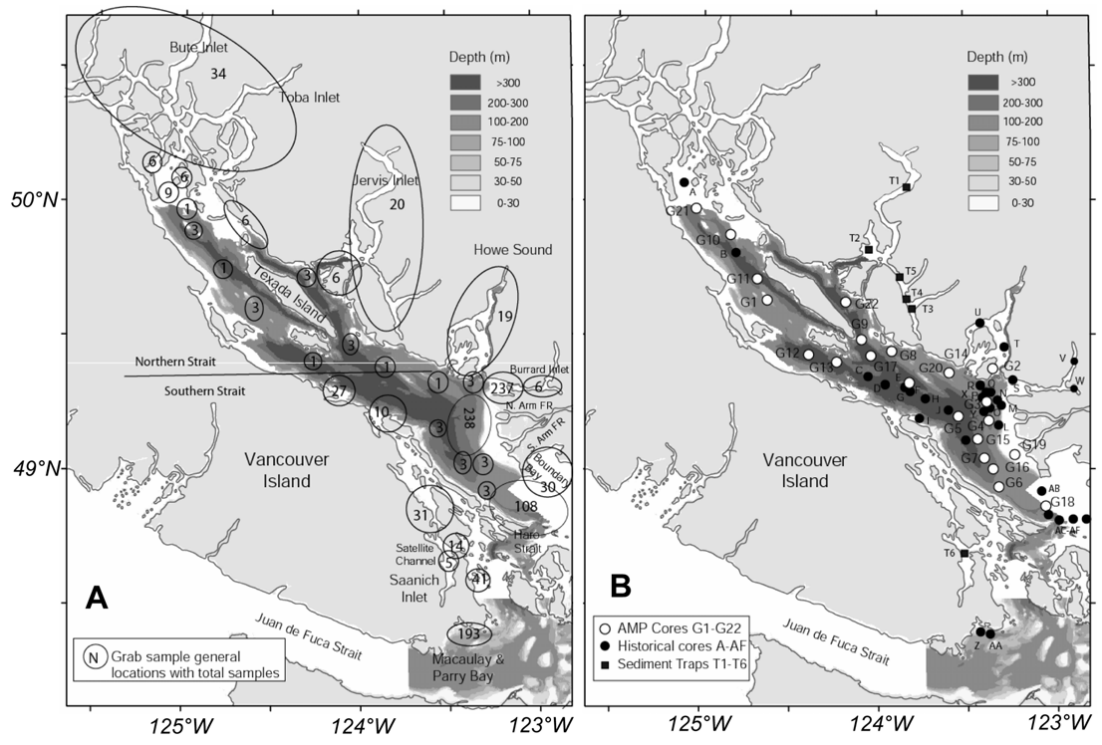
Sampling region (Strait of Georgia) showing general areas and number of benthic invertebrate samples along with bottom bathymetry based on multibeam data (a) (courtesy of Natural Resources Canada). The indicated boundary between the Northern and Southern Straits relates to the limitations of influence to sediments from the Fraser River discharge; and b) Core and sediment trap locations for organic flux measurements (see Table S2 for sources and dates of cores).

Direct measurements of production in marine benthos are extremely time-consuming and difficult to collect on the scale required to understand regional or global processes, resulting in a shift towards the use of empirical or modeled estimates of production relative to biomass (P/B) using community data [6]–[10]. Although such models may be inefficient for estimating the production of a single population, the potential error of estimation declines greatly when averaged over multi-species communities [7], thus making them practical for this use. Several studies have examined regional or global patterns in production and biomass of soft-bottom benthos [9], [11], thereby also providing thorough reviews of the literature, as well as comparisons of the accuracy of different empirical models.

It is typically assumed that organic type and flux to sediments are the most important drivers of benthic biomass and production [2], [6], [12]. Some general comparisons of organic flux to sediments and benthic biomass have been done [11], [13]–[16], sometimes using water depth as a proxy for organic flux [15] since it has been observed in many studies that faunal production, biomass and abundance generally decline with depth in the open ocean [9], [17]–[20] due to a decline in organic flux to sediments [15], [21], [22]. However, in coastal areas, organic flux to sediments can be driven by strong topographic and land-based hydrographic factors (such as river discharges) rather than typical open marine processes. Thus the assumption that organic flux declines in a systematic way with depth is simplistic and misleading in coastal areas, and sometimes in the abyssal ocean [23]. The relationship between sediment type and depth in coastal areas is equally complex [24]. Although benthic production does not appear to respond to sediment type on a global scale [9], this has not been examined in detail for coastal areas.

**Figure 2:**
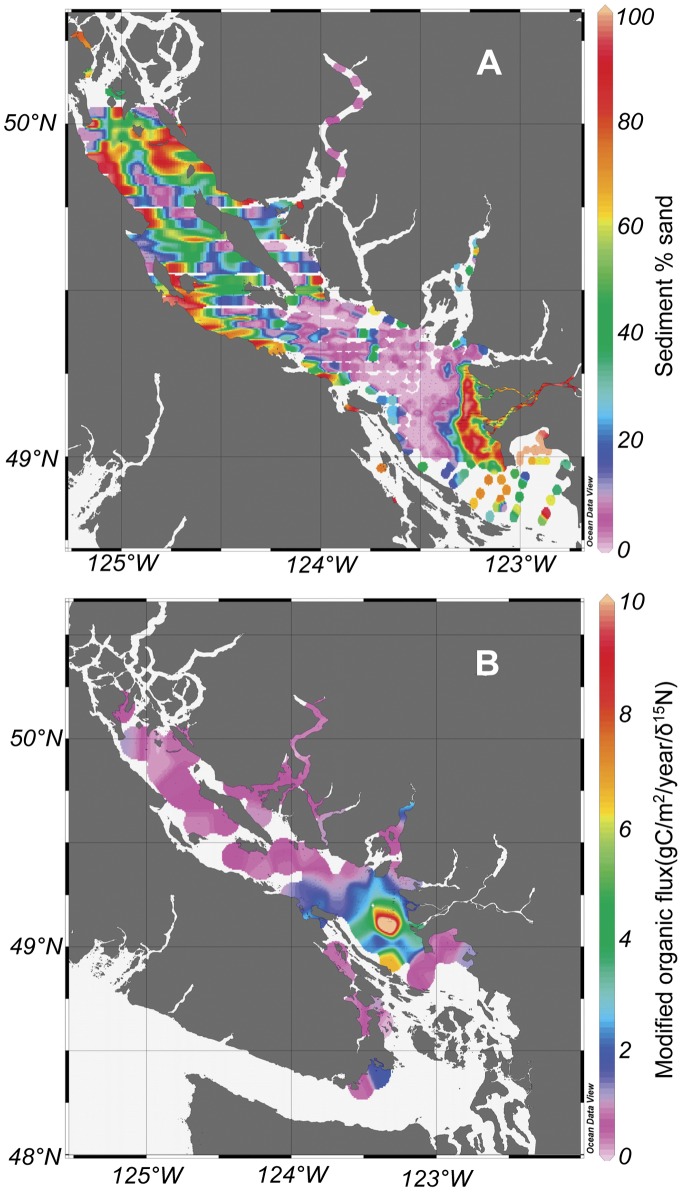
Geographic distribution of habitat variables for the Strait of Georgia. These include a) sediment % sand, and b) modified organic carbon flux (organic carbon flux/del 15N) including values measured from _210_Pb dated cores as well as extrapolated values for locations with biological samples lacking concurrent core data.

In summary, detailed regional analyses of the response of benthic biomass and production to natural environmental conditions are missing. Burd et al. [13] described the distribution of macrobenthic abundance, species richness and faunal types throughout the Strait of Georgia, based on a geographically diverse background database. In this paper, we utilize the same background database from the Strait of Georgia [13] to determine how accurately benthic invertebrate organic biomass and production can be estimated from depth, substrate type and organic flux/lability over a broad geographic region. This study focuses on background areas well distanced from the direct influence of anthropogenic discharges in order to establish natural, baseline estimates of sediment biomass and production.

### Study Area

The Strait of Georgia (northern Salish Sea) has a geographically extensive and hydrographically complex coastline [25], [26]. Including its contiguous fjords, the Strait of Georgia has 3721 km of shoreline [27], and water depths to 700 m. The southern Strait of Georgia (Fig. 1) has strong estuarine circulation related to seasonal input of particulates and freshwater from the Fraser River [3], [28], [29]. Coarser material from the river settles first along the river bank, to a depth of approximately 30 m. Finer material settles at greater depths along the slope, and is primarily transported northward with the prevailing bottom currents. The buoyant plume particles from the river are subject to wider distribution from surface currents, and travel across the Strait to settle out in the deep Southern basin. A moderately shallow sill (about 100 m deep) separates the deeper southern basin of the Strait of Georgia from the northern basin near the south end of Texada Island (Fig. 1), thus preventing bottom-transported particulates from the Fraser River from entering the northern basin [13], [28]. A number of other moderate discharge volume rivers around the Strait also contribute terrestrial inputs to the system, resulting in a complex input of terrestrial and marine organic material [3]. Reviews of benthic conditions and biota in the Strait of Georgia are found in Levings et al. [27] and Burd et al. [28].

## Materials and Methods

The database for the Strait of Georgia includes benthic invertebrate faunal samples along with substrate physical and geochemical data collected from a variety of scientific and monitoring studies. Samples collected range from 0–678 m depth, 0–99% sand and a wide spectrum of organic flux regimes (data sources in Table S1, reference list S1 and [13], [30]). In this study, we use all available subtidal samples from the Strait of Georgia (n = 1067 samples) from background areas that are not proximate to anthropogenic discharges, to obtain estimates of benthic invertebrate biomass and production relative to natural environmental factors. The general location and sample size for these sampling areas is shown in Figure 1. About 90% of the background samples were collected since the year 2000, with all but a handful of locations surveyed once. For the purpose of this study, the data are considered to represent a recent, random spatial and temporal distribution of faunal conditions in the Strait. This database is updated and maintained at the Institute of Ocean Sciences, Sidney, British Columbia (Fisheries and Oceans Canada; contact Brenda.Burd@dfo-mpo.gc.ca.

**Table 1:** Lin’s concordance test between habitat variables.

	modified organic carbon flux	%sand
**%sand**	−.022	
**depth**	.0011	−.188

Field and laboratory methods for all data are described or referenced in Burd et al. [30]. In summary, biological samples were collected using 0.1 m^2^ grab samples (Van Veen or Smith-MacIntyre), screened on 1 mm sieves, initially preserved in formalin and transferred to ethanol for processing. Only samples which included at least 2/3 of the grab volume were included in the database. All taxa were identified to species, or to the lowest possible taxonomic level, and the abundance of adults and sub-adults (juveniles) was enumerated separately. Although all samples were not processed by the same technicians and taxonomists, all surveys followed strict quality control. All faunal samples have associated depths and particle size data. A detailed coding system was used to update and maintain taxonomic consistency across studies and time [31]. The importance of taxonomic consistency in regional databases for large-scale ecological evaluations has been recently highlighted by Vandepitte et al. [32] and Vanden Berghe et al. [33].

**Figure 3:**
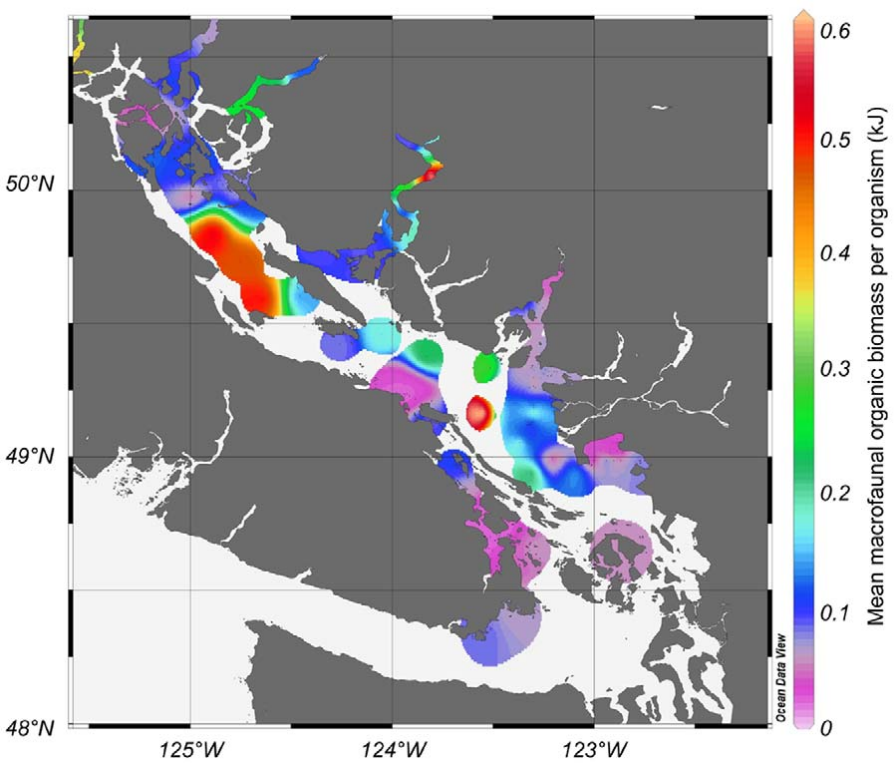
Geographic distribution of mean body mass (kj) per organism in the Strait of Georgia.

Wet-weight biomass values for macrofauna (>1 mm) were estimated from mean species-specific weights in reference collections from each study area. Because wet weights were measured for preserved samples (in 10% buffered formalin for several days prior to transfer to ethanol), it was assumed that biomass shrinkage was either negligible [34], [35], or consistent over the database, since all samples were processed in the same way. Cusson and Bourget [9] used a general conversion factor of 1.2 to compensate for preservation shrinkage (as suggested by Brey [35]). A universal conversion of biomass for preservation was not used in this paper because it would not affect results. However, such a conversion could be important in quantitative modeling of organic carbon budgets in the future. A subset of the macrofaunal samples (N = 64) in the database from a range of depths and substrate types were processed using smaller sieve sizes (0.5 mm, 0.25 mm and 0.125 mm) in addition to the 1 mm seive. All organisms collected in the smaller sieves were separated into major taxonomic groups (nematodes, harpacticoid copepods, foraminifera, juvenile polychaetes, non-harpactoid crustaceans and molluscs), then weighed as a group using a micro-balance with accuracy to 0.01 mg wet weight.

### Environmental Factors

Depths and sediment % sand values were measured concurrently with all biological data (1067 samples). Extensive data of this type for the entire strait were obtained from the Canadian Hydrographic Service and Natural Resources Canada. Bottom bathymetry of the Strait of Georgia based on multibeam data is shown in Figure 1a [36]. The geographic distribution of sediment % sand was compiled from the background database, and from extensive sediment grabs collected by Natural Resources Canada (Fig. 2a).

Analytical calculations for estimating organic carbon flux (sum of buried and oxidized organic material) from _210_Pb dated cores for the Strait of Georgia and surrounding fjords are described in Macdonald et al. [37], with justification for use of the method and comparison with other methods detailed in Johannessen and Macdonald [38]. The cores were all approximately 50 cm long. Immediately on recovery, the cores were sectioned for analysis into 1 cm intervals for the top 10 cm, 2 cm intervals for the next 10 cm and 5 cm intervals for the remainder of the core. A sub-sample from each depth interval was analyzed by Flett Research Ltd., Winnipeg, Canada, for _210_Pb and _226_Ra to be used for radio-dating. The activity of supported _210_Pb was determined as the average of the _226_Ra activity measured at three depths (top, middle, bottom) in each core, from the ingrowths of _226_Rn over at least 4 days. Based on the assumption that bottom waters are always supplied with some oxygen (>2.5 mL L^−1^; [39]), there will be an active benthic community which mixes the surface sediments. Sedimentation and mixing rates in the sediment cores were determined using excess ^210^Pb profiles in sediments together with advective-diffusive models (see Johannessen et al. [40]), and assuming a constant supply of _210_Pb and constant sedimentation rate. The depth of the surface mixed layer in each core was determined by visual measurement from the ^210^Pb profile. The incident flux of organic carbon (OC), the percent OC buried, and the percent OC oxidized, were estimated from the ^210 ^Pb profiles of %OC measured in the sediment cores (see Johannessen et al. [3]). Although not an ideal measure of total sedimenting organic material (which is more accurately measured in bottom sediment traps), this is nevertheless a useful proxy for the amount of organic material that actually remains in sediments (taking resuspension into account) and is thus available for infaunal use. Locations and data for a total of 54 cores from throughout the Strait are shown in Fig. 1b (and see Table S2 for core dates and sources; reference list S1) and are also described in Wright et al. [41], Johannessen et al. [40], [42] and Burd et al. [13] and Carpenter et al. [43]. In addition, detailed measurements of organic carbon flux were available from 6 sediment trap deployments in fjords where no cores were available (Fig. 1b, see Table S2).

A modified organic carbon flux measure was used in this study (described in [13]). This measure weights the organic carbon flux measured from cores by the δ15N ratio. This assumes the lability of settling organic material is dependent on the age and amount of trophic reworking of that material. The higher the δ^15^N value, the less useable the organic material is for most organisms ([13] and references therein). Near-surface stable nitrogen isotopes (δ^15^N) were typically measured in the cores or in nearby surface grabs. Additional isotope data were available from extensive grab sample surveys in the southern Strait of Georgia (unpublished data from Environment Canada’s Ocean disposal program; and Gordon, 1987 [44]). The modification of organic flux was found to be necessary for understanding biological patterns in sediments with naturally high but mostly non-labile organic carbon content [13]. This is also why sediment organic carbon content was not found to be a useful proxy for organic flux to sediments.

Although sediment % sand and depth were available for all samples, organic flux estimates were not always possible, depending on proximity of dated cores or sediment traps. Organic carbon flux and δ^15^N values were assigned to nearby biological sample locations using an exponential variogram. The length scale of the variogram was fitted based on expected scale of variation in the geographic distribution of organic flux rates, using a simple Kriging routine [45] programmed in MATLAB. Source data was filtered to replace clusters of very highly correlated points with their means to make the numerical solution more stable. All extrapolated flux values were examined in detail to ensure that they were rational based on nearby measured values, hydrographic features and topography. Based on this post-hoc examination, it was concluded that flux extrapolations were not possible for some biological sample locations, due to lack of nearby core data. These samples were therefore not included in analyses using flux rates. Particular care was taken to avoid extrapolating in areas where cores were unavailable and unusual influences (such as river discharges) might affect localized flux patterns. Fortunately, numerous cores were available in the SE portion of the main basin of the Strait, which is the area most affected by discharge from the Fraser River, and thus with the widest range in flux rates. After exclusion of biological samples without reasonable flux data, 987 samples were available for data analyses incorporating all three environmental variables concurrently (see Data Analyses).

**Figure 4:**
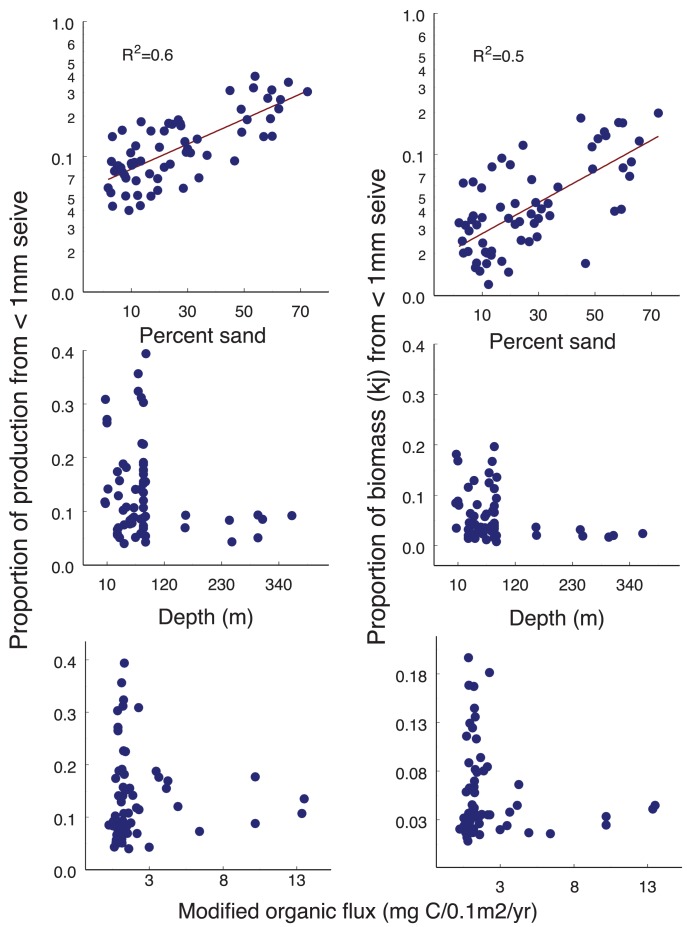
Distribution of the proportion of total invertebrate organic biomass and production contributed by small faunal (<1 mm) organisms, relative to % sand, depth and modified organic flux (N = 65). Only % sand was significantly related to either biomass or production (r^2^ shown on plots, p<0.01; regression coefficients described in results and data shown in Table S4).

### Organic Biomass and Production Estimates

Literature values were used to convert wet weight biomass of all macrofaunal and meiofaunal invertebrate groups to organic carbon weight (see Table S3 for taxa-specific conversions and sources; reference list S1). Where possible, conversion values from Brey [7] were used to align with the production/biomass model estimates (see below). Some of the literature conversion values used were from wet weight to ash-free dry weight. In these cases, after the conversion from wet weight to AFDW, the final conversion used from AFDW to grams of organic carbon (gtoc) was 50% based on consistent estimates from a variety of literature sources [7], [46]–[48].

Dolbeth et al [49] compared a variety of estimation methods for determining annual biomass/production ratios in marine benthic communities and concluded the empirical method of Brey [7], [8] incorporated the most reliable approach. In addition, Cusson and Bourget [9] found that this method closely approximated measured production estimates from direct, classical measurements. For this reason, mean annual P/B ratios were estimated for each macrofaunal taxon in each sample using the empirical formula of Brey [7], which includes depth and bottom temperature, mean body mass (converted to energy units kj – see Table S3) and faunal mobility. From this point forward, organic biomass of invertebrates will be expressed in kj, as used in the original model of Brey [7]. Production and organic biomass values were then summed over all macrofaunal taxa for each sample in the database and an “average” community P/B calculated.

The range in mean annual bottom temperatures for the Strait was about 7–10°C (based on historical hydrographic records at the Institute of Ocean Sciences, Fisheries and Oceans Canada, Sidney, BC Canada). Temperature has been found to be a minor scaling factor in P/B studies by Banse and Mosher [50] and Cusson and Bourget [9] in the range of temperatures typically found in subtidal temperate climates [10]. However, to examine this, P/B values were calculated for all samples for 7, 8 and 10°C. The different temperatures had no appreciable effect on values. Therefore, only P/B ratios calculated using a standard bottom temperature of 7°C are presented in this paper.

Production estimates for the permanent meiofauna (nematodes, copepods) and foraminifera collected on 0.5, 0.25 and 0.125 mm screens had to be calculated separately from macrofauna. Macrofaunal production estimates based on the empirical P/B model of Brey [7] are reasonable for macrobenthos >0.5 mm. Therefore, P/B values for juvenile forms of macrofauna captured in the 0.5 mm sieve were calculated using the model of Brey [7].

**Figure 5:**
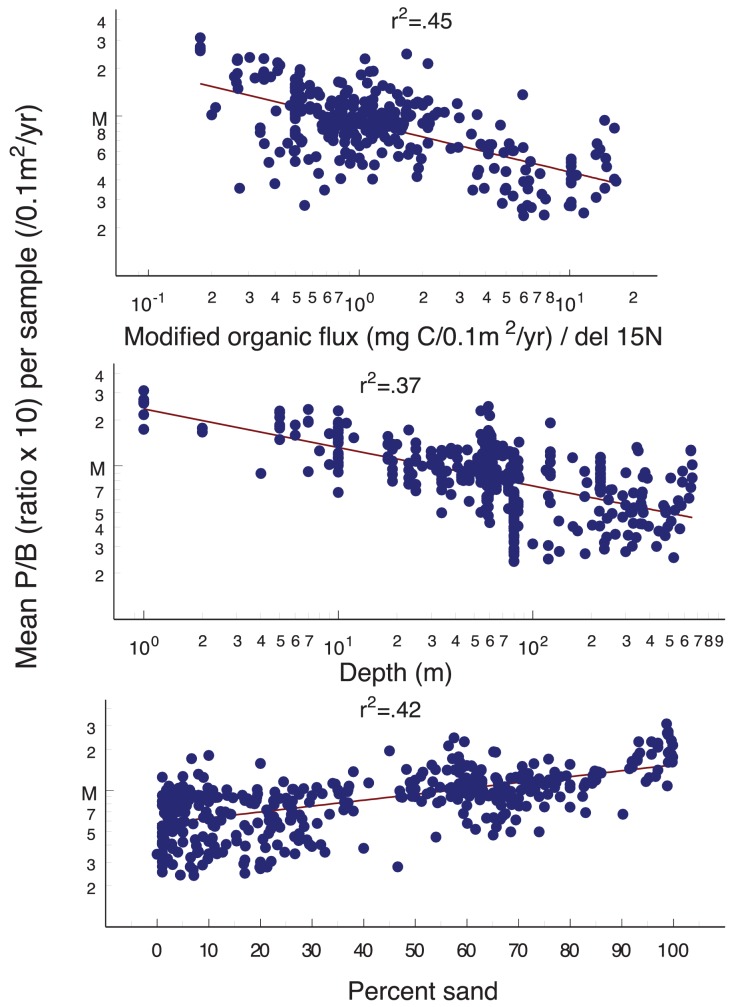
Geographic distribution in the Strait of Georgia of mean total invertebrate production/biomass (P/B) ratio, and values relative to modified organic carbon flux (N = 987), depth and percent sand (N = 1067). Mean values for each sample location and time are shown on figures for visual simplicity and are included in Table S5. Note that the multi-factor regressions (described in results) used only data points for which all three environmental factors were available (N = 987).

Estimating P/B ratios is notoriously difficult for the permanent meiofauna (nematodes, copepods) and for foraminifera, because of limited species-specific information. This lack of information results in a broad range of assumptions and generalizations related to generation times for the remarkable range of sizes and taxonomic diversity of these organisms [11], [51]–[55]. For example, Li et al. [54] and Aller et al. [11] used a P/B ratio of 32 for nematodes of all sizes, whereas Vranken and Heip [52] suggest that values can range between 4 and 69 for different nematode species, depending on the size at maturity. Alongi [55] used a P/B of 91 for nematodes from a variety of environments. In the absence of detailed life span data for individual taxa or size fractions, Heip et al. [56] recommend (with caution) a much lower P/B (10–20) for nematodes of all sizes and 14 for copepods of all sizes. However, we had wet weight biomass measurements for size-specific fractions of permanent meiofauna (0.5 mm, 0.25 mm, 0.125 mm). Therefore, we used empirically-based allometric P/B ratios for permanent meiofauna based on biomass size spectra and sediment respiration rates as per Schwinghamer et al. [57], who estimated annual bulk P/B ratios for permanent meiofauna for a series of sieve size ranges <1 mm as follows; 1.9 for >500 um, 3.8 for >250 um and 7.6 for >125 um).

**Figure 6:**
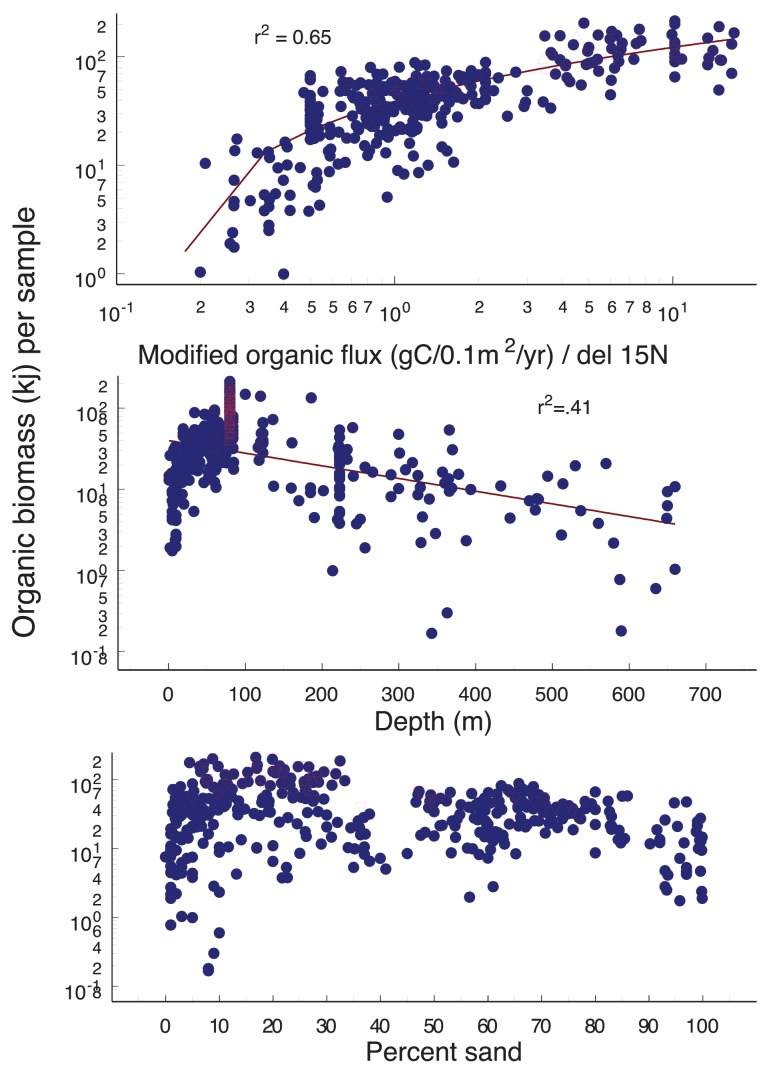
Geographic distribution in the Strait of Georgia of mean total invertebrate biomass, and values relative to modified organic carbon flux (N = 987), depth and percent sand (N = 1067). Mean values for each sample location and time are shown on figures for visual simplicity and are included in Table S5. Note that the multi-factor regressions (described in results) used only data points for which all three environmental factors were available (N = 987). The overlapping red triangles represent samples from near the Fraser River discharge.

**Figure 7:**
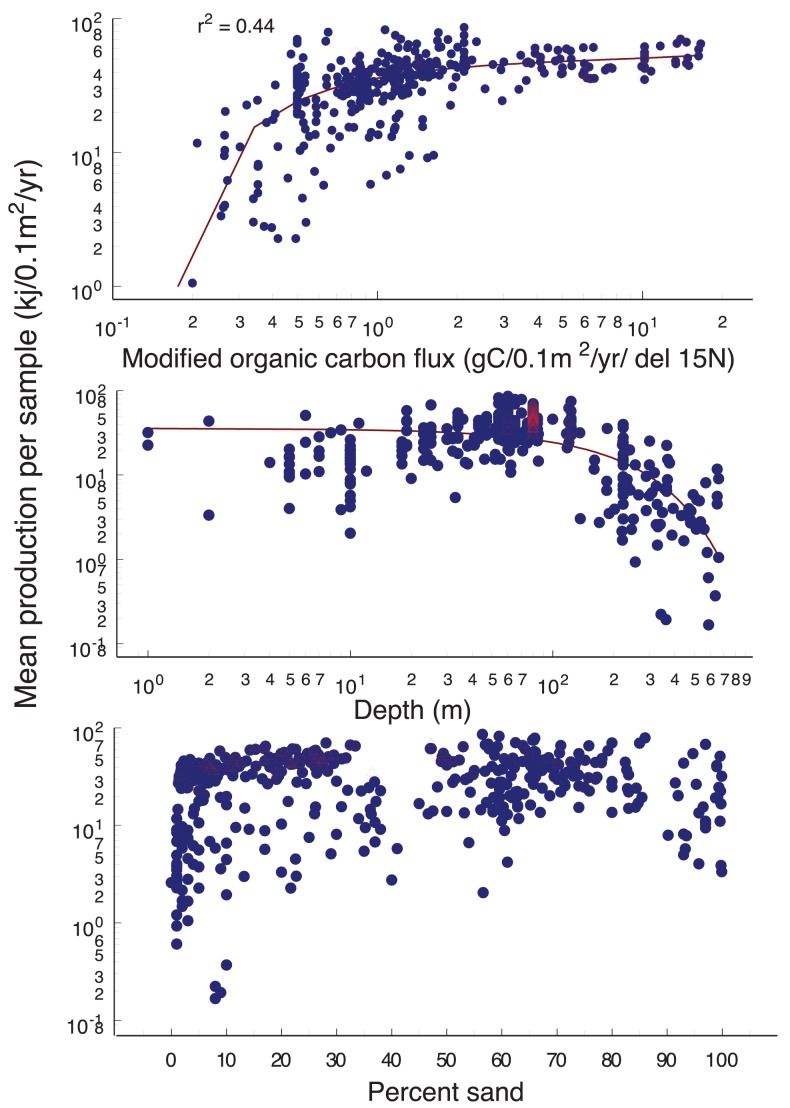
Geographic distribution in the Strait of Georgia of mean total invertebrate production, and values relative to sample modified organic carbon flux (N = 987), depth and percent sand (N = 1067). Mean values for each sample location and time are shown on figures for visual simplicity and are included in Table S5. Note that the multi-factor regressions (described in results) used only data points for which all three environmental factors were available (N = 987). The overlapping red triangles represent samples from near the Fraser River discharge.

### Data Analyses

Lin’s concordance test correlations [58] were used to examine the co-dependence among the 3 environmental variables; a) modified organic carbon flux b) water depth, and c) sediment % sand. The relationships between organic biomass or production and these environmental variables were examined using multi-factor non-linear polynomial regressions [59], in order to examine the cumulative amount of variance explained by the combination of the three environmental variables. Because organic flux was not available for all biological sample locations, the total N for multi-factor regressions was 987. This analysis method also provides measures and plots of the normality and homogeneity of distribution of residuals, to avoid excessive violation of the assumptions of the method.

## Results

Figures 1 and 2a show patterns of depth and substrate % sand in the Strait. The deepest areas typically had fine sediments whereas shallower areas tended to have more variable sediment types (see also Burd et al. [28]). However, this relationship is less clear in the Northern Strait. The finer sediments tended to have a greater range in modified organic flux than sandy sediments, which in general had relatively low organic fluxes (Figs. 2a,b). Modified organic flux (weighted by ^δ15^N) ranged from 0.18 to 16.6 g C/0.1 m^2^/yr, and tended to be highest in the southern basin of the Strait, particularly in the path of buoyant particle plume from the Fraser River [13]. Modified organic flux was generally lowest in the northern basin and the deep mainland fjords (up to 700 m deep; Bute, Toba, Jervis inlets and Howe Sound; see Fig. 1). There were no significant correlations among the environmental variables (Lin’s concordance r values less than 0.19, p>0.05 Table 1). Thus we consider these variables to be independent in the Strait of Georgia.

Figure 3 shows the geographic distribution of mean size (based on organic biomass) of macrofaunal organisms (retained on screens >1 mm) in each sample. The faunal communities varied broadly in richness and faunal abundance [30], from about 15–100 taxa per sample, and 250–15,000 organisms per m^2^. Macrofauna were larger on average in deep locations in the south and north basins of the Strait, and in the deep mainland fjords. Some of the larger organisms were deep, burrowing echinoderms (*Molpadia, Brisaster*), and some larger nemerteans (*Cerebratulus)* and bivalves (*Macoma spp*.). The smallest organisms (on average) were found in shallow, sandy areas, with moderately-sized organisms dominant around the Fraser River discharge. Mean body size is an important factor for understanding patterns in organic biomass and production estimates, since the P/B calculation in Brey [7] is dependent on the mean biomass of each species in each sample.

Small organisms (including permanent meiofauna and some juvenile macrofauna) collected using sieve sizes <1 mm (0.5, 0.250, 0.125 mm) comprised 2–20% of total organic biomass and 1–30% of total production in the samples which could be used for this comparison (64 total – Fig. 4 and Table S4 with sample locations in Figure S1). About ½ of the small organism biomass was in the 0.5 mm sample, which included juvenile macrofauna. It should also be noted that the total juvenile macrofauna captured on both the 1 mm and 0.5 mm sieves comprised a small proportion of the total invertebrate biomass (less than 5%).

**Table 2:** Examples of faunal production estimates from the literature, including habitat type and faunal groups analysed, illustrating ranges measured for infauna from different habitats using different estimation methods (see Cusson and Bourget [9] for a recent and more thorough global review).

Authors	Locale/habitat	Average kj/0.1 m^2^/yr(converted fromoriginal units)	Faunal group	seive size
Cusson and Bourget [9] ++	global all habitats	.034–7295	macrofauna	>.5 mm
Brey and Gerdes [87] *	high antarctic shelf/slope	.4–22	macrofauna	>.5 mm
This study*	Pacific northwest coastal to 700 m	0.15–71	macrofauna	>1 mm
Nilsen et al. [88] *	Norway coastal <350 m	18.5	macrofauna	>1 mm
Thatje and Mutschke [89] *	St of Magellan 8-1139 m	2.85	macrofauna	>.5 mm
Hua et al. [74] *	Bohai Sea	11.7	macrofauna	>0.5 mm
Asmus [78] ++	Northern Wadden Sea tidal flat	115	macrofauna including juveniles	all sizes
Tagliapietra et al. [90] *	Venice lagoon - seagrasses	50.3–138	macrofauna	not given
Tagliapietra et al. [90] *	other studies shallow estuarine	32–276	macrofauna	not given
Probert [79]	Tasmanian shelf	8.3	macrofauna	not given
Dolbeth et al. [49] ++	*Zostera* and non *Zostera* subtropical - intertidal	98.9–480.7	5 species macrobenthos	n/a
Aller et al. [11] ∧	Atlantic shelf/slope- temperate	170.2	macro/meiofauna	>0.3 mm
Danovaro et al. [82] ^∧∧^	seagrass - tropical	34.5–60.7	meiobenthos	<1 mm
This study*	Pacific northwest coastal to 700 m	0.014–28	meiofauna	>.125 mm <1 mm
Ellison [51] ++	intertidal mudflat, UK	178.6	meiobenthos	>.06 mm
Manini et al. [91] ++	Adriatic river plume frontal areas	1506	meiobenthos plus bacteria	<1 mm
Hua et al. [74] *	Bohai Sea	7.5	permanent meiofauna	not given
Probert [79]^	Tasmanian shelf	16.6	meiofauna	not given

++ classical direct measurements (respiration/cohort biomass).

* Empirical models such as Brey [7].

∧ Phylogenetic P/B conversions from literature.

∧∧3 H]-leucine incorporation.

Because there were fewer samples including these smaller organisms, direct patterns in meiofauna biomass and production relative to environmental factors were considered unreliable. Therefore, the relative contribution of the small fauna to total invertebrate biomass and production was compared with the environmental factors. There was a significant exponential increase in the % total invertebrate organic biomass (r^2^ = 0.5, slope = 0.03, intercept 0.02; p<0.01) and production (r^2^ = 0.6, slope = 0.02, intercept = 0.07; p<0.01) made up of these smaller organisms, with increasing % sand (Fig. 4). A multi-factor regression analysis including all three environmental factors showed that virtually none of the variance in proportional organic biomass or production from the smaller organisms could be explained by depth or modified organic flux, and both environmental factors produced non-normal residual distributions. It was therefore concluded that sediment type alone was affecting the relative contribution from the smaller organisms. For this reason, only the single-factor regression equations based on proportion of total invertebrate biomass and production from small organisms versus %sand were used to estimate total (macrofaunal plus meiofaunal) invertebrate organic biomass and production values for all samples in the database that did not originally include data from the smaller sieve sizes. From this point onward, results are shown for this scaled-up total invertebrate organic biomass and production for all size classes.

Summary values for total invertebrate organic biomass and production (mean station values for combined macrofauna/meiofauna for each location and time) are shown on Figs. 5, 6, 7 and included in Table S5 (with sample locations in Figure S1). However, regression analyses described were calculated based on the full complement of available samples, to accurately reflect variability.

Production/Biomass (P/B) for all invertebrates (both macro- and meiofauna) declined with increasing modified organic carbon flux and depth, and increased with increasing percent sand (Fig. 5). The three environmental factors combined explained 69% (r^2^ = 0.69, p<0.01) of the variance in P/B values. P/B values were low near the Fraser River discharge and surrounding deeper areas (where production and biomass were high), low in the deep northern basin and mainland fjords, and highest in the near-shore sandy areas (see Fig. 2), due mainly to dominance by small macrofauna (Fig. 3) and meiofauna in these areas.

Total organic biomass per sample (meio and macrofauna) ranged from 1–253 kj/0.1 m^2^ (Fig. 6). The three environmental variables combined explained 74% of variance in organic biomass (r^2^ = 0.74, p<0.01). The strongest positive relationship was between organic biomass and modified organic carbon flux (r^2^ = 0.65). Organic biomass increased with depth to about 80–120 m, then declined at depths greater than 120 m. Percent sand did not clearly influence total organic biomass. Total organic biomass was greatest near the Fraser River discharge, and was also high across the deeper, southern Strait where the particle plume from the River spreads (Fig. 6; [13] [3]). These areas also have the highest modified organic carbon flux to sediments (Fig. 2b). Moderately high organic biomass values were found near Burrard Inlet (Vancouver Harbour) and along the south shore of Victoria, in Juan de Fuca Strait. The fjords and basin of the northern Strait had the lowest organic biomass values, corresponding with low modified organic carbon flux (Fig. 2b).

Total faunal production ranged from 0.17 to 82 kj/0.1 m^2^/yr (Fig. 7). The combination of the three environmental variables explained 69% (r^2^ = 0.69, p<0.01) of the variance in total production. Production showed a sharp increase with modified organic carbon flux to a certain point (∼3 gC/m2/year/δ^15^ N), then leveled out at higher flux values. Production was fairly uniform to a depth to about 120 m, then declined rapidly below this depth. There was no clear relationship between production and % sand, except that very low production values were only found in the finest sediments, which tended to be from the deepest locations in the Strait. Total production had a similar distribution pattern to organic biomass, except that production values were as high off the Nanaimo River estuary and in Juan de Fuca Strait off Victoria, as in the path of the sediment discharge and buoyant plume from the Fraser River.

## Discussion

Invertebrate organic biomass and production estimates for the range of subtidal ocean bottom conditions examined in this study compare readily with those using similar estimation methods for other temperate coastal seas ([60]; Table 2). A cursory examination of other studies in the literature also shows that there is a wide range in measured values even for similar habitats. This illustrates the difficulty of meta-data comparisons between studies, and the importance of comparing results based on similar methodology [9].

The present study is unique because of the consistent measurement and spatial detail of organic biomass and faunal production estimates over an extensive and highly varied coastal region, as well as the examination of how these biological features are influenced by a suite of environmental factors. Thus we can conclude that for background areas in the Strait of Georgia (without immediate influence of anthropogenic discharges), organic biomass, production and production efficiency in benthic invertebrates can be largely predicted (69–74%) by a combination of depth, substrate type and organic flux/quality. This will allow the identification of areas for which biomass and production are depressed, either due to unusual or changing natural influences, or anthropogenic influences of concern.

The variation in biomass and production over the Strait of Georgia is striking, and reflects the complex hydrography and topography of this coastal sea. Much of the complexity of the Strait of Georgia is driven by the input of organic-rich, fine sediment at depth by the Fraser River in the Southern Strait, and the subsequent restrictions on the passage of this material to the North. This phenomenon results in the lack of basin-wide correlations between the 3 major environmental variables (Table 1). We therefore assume modified organic flux, depth and sediment type are affecting sediment biota relatively independently.

As expected, modified organic flux was the single most important predictor of both organic biomass and production (see also [9], [61]). Depth and substrate type were secondary modifiers, integrating a host of other environmental factors that cannot be systematically accounted for in this type of analysis (bottom currents, habitat stability, seasonal variability in temperature, salinity, etc.). In the deep, northern basin and mainland fjords, low organic biomass was clearly related to low modified organic flux (see also [13], [17], [18], [20]), but may also have been related to spatial and temporal patchiness in food availability. This is a critical factor in faunal structure and recruitment [62]. Declining biomass and production below 200 m may also have been related to loss of bivalves and several other macro-faunal groups as noted in Burd et al. [13]. Furthermore, deep coastal basins often have reduced oxygen levels, resulting in low faunal diversity and biomass [63]–[65]. The deep, mainland fjords and the northern basin of the Strait of Georgia often experience such conditions [28], [66], [67]. The effects of patchiness in food input, food quality [16] and oxygen minimum patterns and duration [16], [68] on infaunal communities in deep coastal areas are all topics that require further study.

Small organisms (<1 mm) are important contributors to the overall organic biomass and production discussed above, particularly in sandy sediments. Their estimated total production is in line with ranges for temperate coastal areas where similar assumptions have been made about meiofaunal biomass and P/B values [11], [47], [57], [69]–[74]. In this study, the proportion of total biomass or production made up of the fauna <1 mm increased exponentially with increasing percent sand. Heip et al. [56] also note in their review that meiofauna become relatively rare in sediments with mean grain size <300 um, which suggests that larger sediment pore spaces facilitate the interstitial forms [75]. Conversely, the relative production from fauna <1 mm was not affected by modified organic flux or depth, a finding shared by other researchers [76], [77]. The conclusion from this study is that meiofauna are more important contributors to organic biomass and production in sandy sediments than in silty ones.

Empirical estimates of P/B are strongly affected by the size spectrum of organisms in the fauna, since smaller species tend to have higher metabolic rates and therefore P/B ratios than larger, longer-lived forms [7]. Production/biomass ratios were therefore highest in shallow, sandy locations, because mean biomass of macrofauna was lowest and the proportion of fauna <1 mm was highest in these areas. The shallow, sandy locations also experienced the highest production efficiencies (production/organic flux up to 55% - not shown) in the Strait. The maximum production efficiencies found in this study seem remarkable, in comparison with production efficiencies estimated for shallow, coastal benthos by Asmus [78], and for deeper Tasmanian shelf benthos by Probert [79]. This high production efficiency may also be related to high bottom currents, which can result in considerable organic material being kept in suspension. We know that suspensivores and facultative deposit/suspension feeders are prominent in these areas [80]. These filter feeders may be metabolizing suspended material not accounted for in sediment accumulation estimates from cores, and therefore unavailable to surface and sub-surface deposit feeders [81].

The lowest P/B values and production efficiencies were found in the deep northern basin of the Strait and the mainland fjords, which tended to have a low proportional biomass of fauna <1 mm.

Danovaro et al. [82] describe a general reduction in ecosystem efficiency (biomass and production) at low organic flux rates in the deep-sea. Other studies have suggested that benthic invertebrate P/B ratios tend to decrease with increasing depth [9], [19], which we speculate is related to patchy or poor quality organic flux to sediments. This could affect infaunal recruitment and thus the size distributions of macro- and meio-faunal organisms [83]. It seems, therefore, that low P/B and production efficiencies tend to accompany low organic biomass, production and organic flux rates in the Strait of Georgia.

Organic biomass and production were expectedly high near the Fraser River discharge, where seasonal inputs of particulates and freshwater result in the highest particulate and organic flux measured throughout the Strait [3], [28], [29]. Because the highest range in organic flux and biomass values is from this one general area, care must be taken in interpreting the full range of organic flux responses. In spite of this, the lower flux samples from this area show considerable overlap with other similar flux regions in the Strait (Figs. 6,7), and the general biomass/production patterns evident in the river discharge area are consistent with the rest of the Strait. Organic biomass shows a relatively steady increase with increasing modified organic flux. However, although production increased in a similar way, it leveled off at moderate modified organic flux rates (∼3gC/m^2^/yr) and did not increase at the higher flux rates found near the Fraser River discharge. This suggests that there is a maximum organic flux that the benthic community can utilize. As a consequence of this limitation of benthic production, P/B ratios were lowest near the Fraser River discharge, suggesting that the benthic organisms there are unable to utilize most of the considerable organic flux present.

Limitations to production within the invertebrate benthos have been described [47], and could be related to predation from mobile organisms rarely caught in grabs [84], [85], or to any factors which tend to increase the mean organism size in communities, such as size selective predation or limitation of recruitment due to competition for space. Alternatively, in high flux areas near the Fraser River discharge in the Strait of Georgia, high inorganic flux results in rapid burial of useable organic material [13], [28] and low sediment organic content (about 1% total organic carbon). Dinn [86] suggested from a study of contaminant uptake in fauna that deposit-feeders in this region must process more material to get the same organic content in their diet as their counterparts in a lower flux area. Thus the high sediment flux rates near the Fraser River may actually be limiting rates of organic consumption and thus production in the benthos.

The patterns described above illustrate the complexity of biological functioning in the Strait of Georgia sediments. The considerable variability in invertebrate biomass and production of sediments found in this complex coastal sea could not be understood or predicted without detailed knowledge of environmental conditions. Organic biomass in the Strait of Georgia is related primarily to food input and quality. However, the limitation to production at the highest modified organic flux levels in the discharge region of the Fraser River, as well as the dichotomous response of production to low modified organic flux in shallow, sandy sediments versus deep, silty ones means that production cannot be adequately explained by food input alone. Substrate type and depth are also important modifiers of production, along with other unmeasured biological and environmental factors. Other factors which are not taken into account in this paper, but which may partially explain the remaining ∼30% of variability in invertebrate production and organic biomass of sediments, include 1) estimation errors, 2) the relative proportion of inorganic flux, 3) temporal patchiness of bottom oxygen and organic flux to sediments, 4) patterns in suspended organic material, and 5) biological interactions such as predation and competition in high biomass areas. The results of this study suggest that it may be possible to do first-order estimates of basin-wide organic carbon inventories and rates of organic carbon turnover in sediment biota for this coastal region.

## Supporting Information

Figure S1 **General sampling regions for Tables S4, S5 biomass lists.**
(DOC)

Table S1 **Data sources, depths and number of samples for all general sample locations shown in** **Fig. 1a**.
(DOC)

Table S2 **List of AMP cores (GVRD 1–22) and historical cores (A-AF), including original published names and coded labels used in** **Fig 1b** **, year of collection.** Core locations are shown in Fig. 1b. Note that T1–T6 were near-bottom sediment trap measurements of organic carbon flux.
(DOC)

Table S3 **Literature conversion values and sources for wet weight to % organic carbon prior to Production/Biomass calculations from the model of Brey (2001).** The conversion from g organic carbon to energy units, as required in the production model used was 46 kj/g organic carbon. [S33, S43, S32, S38]
(DOC)

Table S4 **Listing of meiofaunal biomass and production values, as shown in** **Figure 4** **.** General locations for samples are listed (zone) and cross-referenced to Figure S1.
(DOC)

Table S5 **Summary (means for all sample locations and time) organic biomass and production measurements for macrofaunal (>1 mm) organisms, along with estimates (from the exponential regression with %sand) for small faunal (<1 mm) for each location – Estimated totals are combined for all invertebrate organisms, as shown in** **Figs. 5**, **6**, **7**. Environmental variables are also included. Note that modified organic flux values were not available for all samples. The general sampling zone is cross-referenced to Figure S1.
(DOC)

Reference List S1 (DOC)

## References

[1] Johannessen SC, Macdonald RW, Burd B, van Roodselaar A (2008). Biogeochemical cycling in the Strait of Georgia.. Marine Environmental Research.

[2] Smith C, De Leo F, Bernardino A (2008). Abyssal food limitation, ecoystems structure and climate change.. Trends in Ecology and Evolution.

[3] Johannessen SC, Macdonald RW, Paton DW (2003). A sediment and organic carbon budget for the greater Strait of Georgia.. Estuarine, Coastal and Shelf Science.

[4] Gobas F, Harrad S (2001). Assessing bioaccumulation factors of persistent organic pollutants in aquatic food-chains..

[5] Gobas F, Pasternak J, Lien K, Duncan R (1998). Development & Field-Validation of a multi-media exposure assessment model for waste load allocation in aquatic ecosystems: application toTCDD and TCDF in the Fraser River watershed.. Environmental Science and Technology.

[6] Tumbiolo M, Downing J (1994). An empirical model for the prediction of the secondary production of marine benthic invertebrates.. Marine Ecology Progress Series.

[7] Brey T (2001). Population dynamics in benthic invertebrates. A virtual handbook.. Bremerhaven: Alfred Wegener Institute.

[8] Brey T (2004). Empirical relations in aquatic populations.. In: Population dynamics in benthic invertebrates: a virtual handbook. Bremerhaven: Alfred Wagner Institute..

[9] Cusson M, Bourget E (2005). Global patterns of macroinvertebrate production in marine benthic habitats.. Marine Ecology-Progress Series.

[10] Tagliapietra D, Cornello M, Pessa G (2007). Indirect estimation of benthic secondary production in the Lagoon of Venice (Italy).. Hydrobiologia.

[11] Aller JY, Aller RC, Green MA (2002). Benthic faunal assemblages and carbon supply along the continental shelf/shelf break-slope off Cape Hatteras, North Carolina.. Deep-Sea Research Part II-Topical Studies in Oceanography.

[12] Berkenbusch K, Probert PK, Nodder SD (2011). Comparative biomass of sediment benthos across a depth transect, Chatham Rise, Southwest Pacific Ocean.. Marine Ecology Progress Series.

[13] Burd BJ, Macdonald RW, Johannessen SC, van Roodselaar A (2008). Responses of subtidal benthos of the Strait of Georgia, British Columbia, Canada to ambient sediment conditions and natural and anthropogenic depositions.. Marine Environmental Research.

[14] Grebmeier J, McRoy C, Feder H (1988). Pelagic-benthic coupling on the shelf of the northern Bering and Chukchi Seas. I. Food supply source and benthic biomass.. Marine Ecology Progress Series.

[15] Johnson N, Campbell J, Moore T, Rex M, Etter R (2007). The relationship between the standing stock of deep-sea macrobenthos and surface production in the western North Atlantic.. Deep Sea Research I.

[16] Pusceddu A, Gambi C, Zeppilli D, Bianchelli S, Danovaro R (2009). Organic matter composition, metazoan meiofauna and nematode biodiversity in Mediterranean deep-sea sediments.. Deep Sea Research II.

[17] Vinogradov M, Tseitlin V, Rowe GT (1983). Deep-sea pelagic domain (aspect of bioenergetics)..

[18] Shirayama Y (1984). The abundance of deep-sea meiobenthos in the western Pacific in relation to environmental factors.. Oceanologica Acta.

[19] Jumars P, Banse K (1989). Benthos and its interaction with bottom boundary layer processes chapter 9 in coastal oceanography of Washington and Oregon.. Elsevier Oceanography Series.

[20] Vanaverbeke J, Soetaert K, Heip CHR, Vanreusel A (1997). The metazoan meiobenthos along the continental shelf of the Goban Spur (NE Atlantic).. Journal of Sea Research.

[21] Rex M, Etter R, Morris J, Crouse J, McClain C (2006). Global bathymetric patterns of standing stock and body size in the deep-sea benthos.. Marine Ecology Progress Series.

[22] Smith C, Berelson W, Demaster D, Dobbs F, Hammond D (1997). Latitudinal variations in benthic processes in the abysall equatorial Pacific: control by biogenic particle flux.. Deep Sea Research II.

[23] De Leo F, Smith C, Rowden A, Bowden D, Clark M (2010). Submarine canyons: hotspots of benthic biomass and productivity in the deep sea.. Proceedings of the Royal Society B: Biological Sciences.

[24] Snelgrove PVR, Butman CA (1994). Animal sediment relationships revisited - cause versus effect.. Oceanography and Marine Biology.

[25] Thomson RE (1981). Oceanography of the British Columbia Coast.. Canadian Special Publication of Fisheries and Aquatic Sciences.

[26] Davenne E, Masson D (2001). Water properties in the Straits of Georgia and Juan de Fuca.. Fisheries and Oceans Canada www-sci.pac.dfo-mpo.gc.ca/osap/projects/straitofgeorgia/JdFG_e.pdf.

[27] Levings CD, Foreman RE, Tunnicliffe VJ (1983). Review of the benthos of the Strait of Georgia and contiguous fjords.. Canadian Journal of Fisheries and Aquatic Sciences.

[28] Burd BJ, Barnes PAG, Wright CA, Thomson RE (2008). A review of subtidal benthic habitats and invertebrate biota of the Strait of Georgia, British Columbia.. Marine Environmental Research.

[29] Hill PR, Conway K, Lintern DG, Meulé S, Picard K (2008). Sedimentary processes and sediment disperal in the southern Strait of Georgia, B.C., Canada.. Marine Environmental Research.

[30] Burd BJ, McGreer E, Taekema B, Macdonald TA (2009). Utility of large regional databases for understanding abundance and diversity characteristics of natural marine soft substrate fauna.. Canadian Technical Report of Fisheries and Aquatic Sciences.

[31] Macdonald TA, Burd B, Macdonald VI, van Roodselaar A (2010). Taxonomic and feeding guild classification for the marine benthic macroinvertebratesof the Strait of Georgia, British Columbia.. Canadian Technical Report of Fisheries and Aquatic Sciences 2874: 63 p.

[32] Vandepitte L, Vanhoorne B, Kraberg A, Anisimova N, Antoniadou C (2010). Data integration for European marine biodiversity research: creating a database on benthos and plankton to study large-scale patterns and long-term changes Hydrobiologia.

[33] Vanden Berghe E, Claus S, Appeltans W, Faulwetter S, Arvanitidis C (2009). MacroBen integrated database on benthic invertebrates of European continental shelves: a tool for largescale analysis across Europe.. Hydrobiologia.

[34] Leuven R, Brock TCM, Vandruten HAM (1985). Effects of preservation on dry-free and ash-free dry-weight biomass of some common aquatic macroinvertebrates.. Hydrobiologia.

[35] Brey T (1986). Formalin and formaldehydy depot chemicals - effects on dry-weight and ash-free dry weight of two marine bivalve species Meeresforschung-Reports on Marine Research.

[36] Barrie J, Currie R, Kung R (2008). Georgia Basin geohazards initiative, Fraser Delta - surficial sediment distribution and human impact.. Geological Survey of Canada.

[37] Macdonald RW, Johannessen SC, Gobeil C, Wright CA, Burd BJ (2008). Sediment redox tracers in the Strait of Georgia sediments - can they inform us of the loadings of organic carbon from municipal wastewater?. Marine Environmental Research.

[38] Johannessen SC, Macdonald RW (2012). There is no 1954 in that core! Interpreting sedimentation rates and contaminant trends in marine sediment cores.. Marine Pollution Bulletin.

[39] Masson D (2002). Deep water renewal in the Strait of Georgia.. Estuarine and Costal Shelf Science.

[40] Johannessen SC, Macdonald RW, Eek MK (2005). Historical trends in mercury sedimentation and mixing in the Strait of Georgia, Canada.. Environmental Science and Technology.

[41] Wright CA, Johannessen SC, Macdonald RW, Burd BJ, Hill PR (2008). The Strait of Georgia Ambient Monitoring Program, Phase I 2002–2007: Sediment and Benthos. Canadian Data Report of Fisheries and Aquatic Sciences.. vi +112 p.

[42] Johannessen SC, O’Brien MC, Denman KL, Macdonald RW (2005). Seasonal and spatial variations in the source and transport of sinking particles in the Strait of Georgia, British Columbia, Canada.. Marine Geology.

[43] Carpenter R, Bennett JT, Peterson ML (1981). Pb^210^ activities in and fluxes to sediments of the Washington continental shelf and slope.. Geochimica Et Cosmochimica Acta.

[44] Gordon K (1997). Sedimentary tracers of sewage inputs to the southern Strait of Georgia [MSc.]. Vancouver, BC: University of British Columbia.. 217 p.

[45] Isaaks E, Srivastava R (1989). An Introduction to Applied Geostatistics: Oxford University Press.. 592 p.

[46] Ricciardi A, Bourget E (1998). Weight-to-weight conversions factors for marine benthic macroinvertebrates.. Marine Ecology Progress Series.

[47] Galeron J, Sibuet M, Mahaut M-L, Dinet A (2000). Variation in structure and biomass of the benthic communities at three contrasting sites in the tropical Northeast Atlantic.. Marine Ecology Progress Series.

[48] Clarke A (2008). Ecological stoichiometry in six species of Antarctic marine benthos Marine Ecology Progress Series.

[49] Dolbeth M, Lillebo A, Cardosa P, Ferreira S, Pardal M (2005). Annual production of estuarine fauna in different environmental conditions: An evaluation of the estimation methods.. Journal of Experimental Marine Biology and Ecology.

[50] Banse K, Mosher S (1980). Adult body mass and annual production/biomass relationships of field populations.. Ecological Monographs.

[51] Ellison R (1984). Foraminifera and meiofauna on an intertidal mudflat, Cornwall, England: Populations; respiration and secondary production, and energy budget.. Hydrobiologia.

[52] Vranken G, Heip CHR (1986). The productivity of marine nematodes, in: Muus, K. (Ed.) (1986). Proceedings of the 20th European Marine Biology Symposium: Nutrient Cycling. Processes in Marine Sediments, Hirtshals, Denmark, 9–13 September 1985.. Ophelia: International Journal of Marine Biology.

[53] Vranken G, Herman P, Vincx M, Heip C (1986). A re-evaluation of marine nematode productivity.. Hydrobiologia.

[54] Li J, Vincx M, Herman P (1997). Carbon flows through meiobenthic nematodes in the Westerschelde Estuary.. Fundamentals of Applied Nematology.

[55] Alongi D (1998). Coastal ecosystem processes. Boca Raton: CRC Press.. 419 p.

[56] Heip C, Brandt A, Gattuso J-P, Antia A, Berger W, Wefer G, Lamy F, Mantoura F (2003). Ecosystem functioning and marine biodiversity..

[57] Schwinghamer P, Hargrave B, Peer D, Hawkins CM (1986). Partitioning of production and respiration among size groups of organisms in an intertial benthic community.. Marine Ecology-Progress Series.

[58] Lin L-K (2000). A note on the concordance correlation coefficient.. Biometrics.

[59] Wessa P (2010). Free Statistics Software, Office for Research Development and Education, version 1.1.23-r6..

[60] Dekker R (1989). The macrozoobenthos of the subtidal western Dutch Wadden Sea. 1. Biomass and species richness.. Netherlands Journal of Sea Research.

[61] Danovaro R, Gambi C, Mirto S (2002). Meiofaunal production and energy transfer efficiency in a seagrass Posidonia oceanic bed in the western Mediterranean.. Marine Ecology Progress Series.

[62] Soltwedel T, Pfannkuche O, Thiel H (1996). The size structure of deep-sea meiobenthos in the north-eastern Atlantic: Nematode size spectra in relation to environmental variables.. Journal of the Marine Biological Association of the United Kingdom.

[63] Llansó R, Aasen S, Welch K (1998). Marine Sediment Monitoring Program: II. Distribution and Structure of Benthic Communities in Puget Sound 1989–1993. Olympia, Washington: Washington State Department of Ecology: Environmental investigations and laboratory services program. Publication No. 98–328 Publication No. 98–328.. 114 p.

[64] Rosenberg R (2001). Marine benthic faunal successional stages and related sedimentary activity.. Scientia Marina.

[65] Seitz R, DAuer D, Llansó R, Long W (2009). Broad-scale effects of hypoxia on benthic community structure in Chesapeake Bay, USA.. Journal of Experimental Marine Biology and Ecology.

[66] Stucchi D (2009). Long Term Trends in Deep Water Properties of BC Inlets.. Institute of Ocean Sciences, Sidney BC: Fisheries and Oceans Canada.

[67] Johannessen S, Macdonald R (2009). Effects of local and global change on an inland sea: the Strait of Georgia, British Columbia, Canada.. Climate Research.

[68] Diaz R, Rosenberg R (2008). Spreading dead zones and consequences for marine ecosystems.. Science.

[69] Gerlach S (1971). On the importance of marine meiofauna for benthos communities.. Oecologia.

[70] Gerlach S (1978). Food-chain relationships in subtidal silty sand marine sediments and the role of meiofauna in stimulating bacterial productivity.. Oecologia.

[71] Beukema J, Dankers N, Kühl H, Wolff W (1983). Quantitative data on the benthos of the Wadden Sea proper..

[72] Witte J, Zijlstra J (1984). The meiofauna of a tidal flat in the western part of the the Wadden Sea and its role in the benthic ecosystem.. Marine Ecology Progress Series.

[73] Rowe G, Wei C, Nunnally C, Haedrich R, Montagna P (2008). Comparative biomass structure and estimated carbon flow in food webs in the deep Gulf of Mexico.. Deep-Sea Research II.

[74] Hua E, Zhou H, Zhang Z, Yu Z (2010). Estimates of autumntime benthic secondary production in Laizhou Bay and adjacent Bohai sea waters.. Journal of Ocean University of China (English).

[75] Wigley R, McIntyre A (1964). Some quantitative comparisons of offshore meiobenthos and macrobenthos sou of Martha’s vineyard.. Limnology and Oceanography.

[76] Coull B (1999). Role of meiofauna in estuarine sof-bottom habitats.. Australian Journal of Ecology.

[77] Altenbach A, Struck U (2001). On the coherence of organic carbon flux and benthic foraminiferal biomass.. Journal of Foraminiferal Research.

[78] Asmus H (1982). Field measurements on respiration and secondary production of a benthic community in the northern Wadden Sea.. Netherlands Journal of Sea Research.

[79] Probert P (1986). Energy transfer through the shelf benthos off the west coast of South Island, New Zealand.. New Zealand Journal of Marine and Freshwater Research.

[80] Macdonald T, Burd B, van Roodselaar A (2011). Facultative feeding and consistency of trophic structure in marine soft-bottom macrobenthic communities.. Marine Ecology Progress Series.

[81] Marr M, Hansen J (2011). Increasing temperatures change pelagic trophodynamics and the balance between pelagic and benthic secondary production in a water column model of the Kattegat.. Journal of Marine Systems.

[82] Danovaro R (2008). Exponential decline of deep-sea ecosystem functioning linked to benthic biodiversity loss.. Current Biology.

[83] Warwick RM (1984). Species size distributions in marine benthic communities.. Oecologia.

[84] Evans S (1983). Production, predation and food nich segregation in a marine shallow soft-bottom community.. Marine Ecology Progress Series.

[85] Danovaro R, Scopa M, Gambi C, Fraschetti S (2007). Trophic importance of subtidal metazoan meiofauna: evidence from in situ exclusion experiments on soft and rocky substrates.. Marine Biology.

[86] Dinn P (2011). Receiving environment shapes transport and bioaccumulation of polybrominated diphenyl ethers near two submarine municipal outfalls.. MSc thesis University of Victoria School of Earch and Ocean Sciences.

[87] Brey T, Gerdes D (1998). High Antarctic macrobenthic community production.. Journal of Experimental Marine Biology and Ecology.

[88] Nilsen M, Pedersen T, Nilssen EM (2006). Macrobenthic biomass, productivity P/B and production in a high-latitude ecosystem, North Norway.. Marine Ecology Progress Series.

[89] Thatje S, Mutschke E (1999). Distribution of abundance, biomass, production and productivity of macrozoobenthos in the sub-Antarctic Nagellan Province (South America).. Polar Biology.

[90] Tagliapietra D, Cornello M, Pessa G (2007). Indirect estimation of benthic secondary production in the Lagoon of Venice (Italy).. Hydrobiologia.

[91] Manini E, Danovaro R, Fabiano M (2002). Benthic-pelagic coupling in front system areas of the Northern Adriatic Sea: Analysis of the carbon budgets.. Chemistry and Ecology.

