# Supplementary material for: Towards Predicting Basin-Wide Invertebrate Organic Biomass and Production in Marine Sediments from a Coastal Sea
Source: PLoS One. 2012 Jul 6;7(7):e40295. doi: 10.1371/journal.pone.0040295 (PMC3391270; doi:10.1371/journal.pone.0040295)
Supplement: Figure S1 — General sampling regions for Tables S4, S5 biomass lists. (DOC) [file pone.0040295.s001.doc]

Supporting Information Figure S1.
