# Supplementary material for: Towards Predicting Basin-Wide Invertebrate Organic Biomass and Production in Marine Sediments from a Coastal Sea
Source: PLoS One. 2012 Jul 6;7(7):e40295. doi: 10.1371/journal.pone.0040295 (PMC3391270; doi:10.1371/journal.pone.0040295)
Supplement: Table S1 — Data sources, depths and number of samples for all general sample locations shown in Fig. 1a . (DOC) [file pone.0040295.s002.doc]

Supporting Table S1.

| Region | acronym | Code | Purpose of study | Year | Samples | Depth (m) | References |
| --- | --- | --- | --- | --- | --- | --- | --- |
| Saanich Inlet | SI | 1 | Ambient monitoring for Collaborative project (DFO/Metro) | 2010 | 5 | 5-12 | Burd, unpublished |
| Northern main basin of Strait | Tully | 2 | Ambient monitoring for Collaborative project (DFO/Metro) | 2010 | 6 | 160-300 | Burd, unpublished |
| Boundary Bay – Canada/US border | BB | 3 | Baseline nearshore - DFO | 1986 | 30 | 29-46 | Burd et al. [S1] |
| Ecological Reserve 67, Southern Gulf Islands | ER67 | 4 | Background for pipeline | 1999 | 14 | 60-70 | Burd and Glaholt, [S2] |
| Mainland fjords | Fjords | 5 | Ambient for deep fjords | 1988-1990 | 42 | 221-634 | Burd and Brinkhurst [S3] |
| Southeast Strait of Georgia | Iona | 6 | Ambient for outfall | 2001-2010 | 238 | 80 | Lynch et al. [S4] |
| Southeast Vancouver Island | Manley Landing | 7 | Background for pipeline landing | 2001 | 25 | 1-25 | Burd and Glaholt [5]; Seacology and Tera [S6] |
| Strait of Georgia | EEM | 8 | EEM Ambient for pulp mills | 2002-2006 | 34 | 30-135 | http://www.cofi.org/library_and_resources/publications/environmental_energy/pdf/cofi2000.pdf |
| Howe Sound | Brittania | 9 | Brittania Beach AMD | 2000 | 7 | 5-20 | G3 [S7] |
| Gorge Harbour, Village Bay, Saltspring Island | GH, VB | 10 | Reference related to oyster farm biodeposition | 2005 | 12 | 13-37m | Barnes [S8] |
| Outer Burrard Inlet | LG | 11 | Ambient for Lions Gate outfall | 2002-2010 | 237 | 55-75 | McPherson et al. [S9] |
| Northern Strait of Georgia; Johnstone St | FF | 12 | BCMOE Fish farm monitoring | 2000-2007 | 18 | 30-100 | Wright et al. [S10-S14]; BC Ministry of Environment, unpublished data |
| Main basin Strait of Georgia | AMP | 13 | Ambient monitoring program: Strait of Georgia | 2003, 2004, 2006, 2007 | 24 | 80-340 | Wright et al. [S15] McPherson et al. [S16] |
| Southern Strait of Georgia | PSAMP | 14 | Puget Sound Ambient Monitoring Program | 1989-2008 | 108 | 20-233 | http://www.ecy.wa.gov/programs/eap/psamp/TemporalMonitoring/Temporal.htm |
| Parry Bay, Juan de Fuca Strait | Macaulay | 15 | CRD Macaulay Point outfall monitoring | 2000-2007 | 193 | 60-70 | http://www.crd.bc.ca/wastewater/marine/reports.htm;  Paine et al. [S17] |
| Southern Gulf Islands | SP | 16 | CRD Saanich Peninsula outfall monitoring | 2004, 2008 | 7 | 30-32 | http://www.crd.bc.ca/wastewater/marine/saanich_peninsula/index.htm |
| East side Saanich Peninsula | Bazan Bay | 17 | Pipeline study for GSX crossing | 2001 | 34 | 10-12 | Glaholt et al. [S18] |
| Nanaimo Harbour | Nhbr | 18 | Background for outfall | 2007 | 27 | 60-70 | Associated Engineering and Lorax Environmental [S19] |
