# Supplementary material for: Towards Predicting Basin-Wide Invertebrate Organic Biomass and Production in Marine Sediments from a Coastal Sea
Source: PLoS One. 2012 Jul 6;7(7):e40295. doi: 10.1371/journal.pone.0040295 (PMC3391270; doi:10.1371/journal.pone.0040295)
Supplement: Table S2 — List of AMP cores (GVRD 1–22) and historical cores (A-AF), including original published names and coded labels used in Fig 1b , year of collection. Core locations are shown in Fig. 1b. Note that T1–T6 were near-bottom sediment trap measurements of organic carbon flux. (DOC) [file pone.0040295.s003.doc]

Supporting Table S2.

| **Original Core name** | **Code**  **(Fig. 2)** | **Year Collected** | **Reference** |
| --- | --- | --- | --- |
| 17 | Q | 1993 | Yunker et al. [S20]; Johannessen et al. [S21,S22] |
| 21 | O | 1993 | Yunker et al. [S20]; Johannessen et al. [S21,S22] |
| 31 | P | 1993 | Yunker et al. [S20]; Johannessen et al. [S21,S22] |
| A | R | 1993 | Yunker et al. [S20]; Johannessen et al. [S21,S22] |
| BB1 | J | 1990 | Macdonald et al. [S23]; Johannessen et al. [S21, S22] |
| BC-2 | G |  | Johannessen et al. [S21, S22] |
| CM-3 | K | 1997 | Johannessen et al. [S21, S22] |
| CM-4 | F | 1997 | Johannessen et al. [S21, S22] |
| FD-12 | L | 1996 | Shang et al. [S24]; Johannessen et al. [S21, S22] |
| FD-22 | M | 1996 | Shang et al. [S24]; Johannessen et al. [S21, S22] |
| FD-3 | N | 1996 | Shang et al. [S24]; Johannessen et al. [S21, S22] |
| HS-1 | T | 1990 | Macdonald et al. [S23]; Johannessen et al. [S21, S22] |
| IND-VI | V | 1994 | Boyd et al. [S25]; Johannessen et al. [S21, S22] |
| PEI | S | 1994 | Boyd et al. [S25]; Johannessen et al. [S21, S22] |
| PM-V | W | 1994 | Boyd et al. [S25]; Yunker et al. [S20]; Johannessen et al. [S21, S22] |
| SC | A | 2001 | Johannessen et al. [S21, S22] |
| TC-1 | U | 1990 | Macdonald et al., 1992; Johannessen et al. [S21, S22] |
| TI | B | 2001 | Johannessen et al. [S21, S22] |
| Vec04A-10 | D | 2004 | Picard et al. [S26] |
| Vec04A-11 | I | 2004 | Picard et al. [S26] |
| Vec04a-15 | C | 2004 | Picard et al. [S26] |
| Vec04A-16 | E | 2004 | Picard et al. [S26] |
| Vec04A-17 | H | 2004 | Picard et al. [S26] |
| IO-400mN | X | 2010 | Dinn et al. [S27] |
| IO-400mS | Y | 2010 | Dinn et al. [S27] |
| CRD-1 | Z | 2010 | Dinn et al. [S27] |
| CRD-2 | AA | 2010 | Dinn et al. [S27] |
| 66 | AB | 1984 | Carpenter et al. [S28] |
| 67 | AC | 1984 | Carpenter et al. [S28] |
| 68 | AD | 1984 | Carpenter et al. [S28] |
| 69 | AE | 1984 | Carpenter et al. [S28] |
| 70 | AF | 1984 | Carpenter et al. [S28] |
| GVRD-1 | G-1 | 2003 | Wright et al. [S15] |
| GVRD-2 | G-2 | 2003 | Wright et al. [S15] |
| GVRD-3 | G-3 | 2002 | Wright et al. [S15] |
| GVRD-4 | G-4 | 2002 | Wright et al. [S15] |
| GVRD-5 | G-5 | 2002 | Wright et al. [S15] |
| GVRD-6 | G-6 | 2003 | Wright et al. [S15] |
| GVRD-7 | G-7 | 2003 | Wright et al. [S15] |
| GVRD-9 | G-9 | 2007 | Wright et al. [S15] |
| GVRD-10 | G-10 | 2007 | Wright et al. [S15] |
| GVRD-11 | G-11 | 2008 | Wright et al. [S15] |
| GVRD-12 | G-12 | 2008 | Wright et al. [S15] |
| GVRD-13 | G-13 | 2008 | Wright et al. [S15] |
| GVRD-14 | G-14 | 2008 | Wright et al. [S15] |
| GVRD-15 | G-15 | 2008 | Wright et al. [S15] |
| GVRD-16 | G-16 | 2008 | Wright et al. [S15] |
| GVRD-17 | G-17 | 2008 | Wright et al. [S15] |
| GVRD-18 | G-18 | 2008 | Wright et al. [S15] |
| GVRD-19 | G-19 | 2008 | Wright et al. [S15] |
| GVRD-20 | G-20 | 2008 | Wright et al. [S15] |
| GVRD-21 | G-21 | 2008 | Wright et al. [S15] |
| GVRD-22 | G-22 | 2008 | Wright et al. [S15] |
| JV3 | T1 | 1989 | *Timothy et al. [S29] |
| JV7 | T2 | 1989 | *Timothy et al. [S29] |
| sc3 | T3 | 1991 | Timothy [S30] |
| sc5.5 | T4 | 1991 | Timothy [S30] |
| sc7 | T5 | 1991 | Timothy [S30] |
| SN9 | T6 | 1989 | *Timothy et al. [S29] |
