# Supplementary material for: Towards Predicting Basin-Wide Invertebrate Organic Biomass and Production in Marine Sediments from a Coastal Sea
Source: PLoS One. 2012 Jul 6;7(7):e40295. doi: 10.1371/journal.pone.0040295 (PMC3391270; doi:10.1371/journal.pone.0040295)
Supplement: Table S3 — Literature conversion values and sources for wet weight to % organic carbon prior to Production/Biomass calculations from the model of Brey (2001). The conversion from g organic carbon to energy units, as required in the production model used was 46 kj/g organic carbon. [S33, S43, S32, S38] (DOC) [file pone.0040295.s004.doc]

Supporting Table S3.

| **General faunal groups** | % organic carbon (from wet weight) | **Reference** |
| --- | --- | --- |
| Actinaria | 6.3 | Galeron et al. [S31] |
| Anthozoa (general) | 6.9 | Brey [S32] |
| Octocorals | 5.87 | Steimle and Terranova [S33] |
| Anemones | 7.46 | Steimle and Terranova [S33] |
| *Cerianthiopsis americanus* | 5.24 | Frithsen et al. [S34] |
| Brachiopoda | 5.43 | Steimle and Terranova [S33] |
| Bryozoa | 3.65 | Ricciardi and Bourget [S35],Clarke [S36], Brey [S32] |
| Bivalvia | 2.75 | Lie [S37], Cauffope and Heymans [S38], Brey [S32] |
| *Clinocardium ciliatum* | 1.5 | Ricciardi and Bourget [S35], Clarke [S36] |
| opistho-shelled | 6.9 | Ricciardi and Bourget [S35], Clarke [S36] |
| opistho-non shelled | 8.58 | Ricciardi and Bourget [S35] |
| Prosobranchia | 3.4 | Rowe [S39] |
| Crustacea (general) | 8 | Brey [S32] |
| Amphipoda | 4.5 | Rowe [S39], Frithsen et al. [S34] |
| *Ampelisca abdita* | 6.48 | Frithsen et al. [S34] |
| *Corophium sp* | 2.03 | Frithsen et al. [S34] |
| *Jassa pelagica* | 7.14 | Frithsen et al. [S34] |
| *Leptocheirus pinguis* | 4.08 | Frithsen et al. [S34] |
| Cirripedia | 1.95 | Brey [S32] |
| Cumacea | 3.75 | Brey [S32] |
| Decapoda | 9 | Brey [S32] |
| Isopoda | 7.1 | Ricciardi and Bourget [S35], Clarke [S36], Brey [S32] |
| Leptostraca | 7.5 | Lie [S38] |
| Mysidacea | 7.75 | Ricciardi and Bourget [S35], Clarke [S36] |
| Ostracoda | 6 | Rudnick et al. [S40] |
| Tanaidacea | 2.9 | Rowe [S39] |
| Miscellaneous crustacea | 8.45 | Brey [S32] |
| Hirudinea | 6.5 | Leuven et al. [S41] |
| Hydrozoa | 2.3 | Rowe [S39] |
| **Echinodermata** |  |  |
| Asteroidea | 6.2 | Brey [S32] |
| Echinoidea | 2.45 | Brey [S32] |
| Holothuroidea | 5.6 | Brey [S32] |
| Ophiuroidea | 4.5 | Brey [S32] |
| Echiura | 5.1 | Rowe [S39] |
| Entoprocta | 3.65 | Ricciardi and Bourget [S35], Clarke [S36], Brey [S32] |
| Hemichordata | 3.8 | Fielman and Target [S42] |
| Kinorhyncha | 11.6 | Brey [S32] |
| Aplacophora | 5.7 | Rowe [S39] |
| Nemertea | 10 | Ricciardi and Bourget [S35], Clarke [S36], Brey [S32] |
| Phoronida | 5.1 | Rowe [S39] |
| Platyhelminthes | 12.6 | Ricciardi and Bourget [S35], Clarke [S36] |
| Oligochaeta | 16.15 | Ricciardi and Bourget [S35], Clarke [S36] |
| Polychaetes (general) | 5.1 | Rowe [S39] |
| Errantiate polychaetes | 8.5 | Ricciardi and Bourget [S35], Clarke [S36], Cauffope and Heymans [S38], Brey [S32] |
| *Nephtys incise* | 7.49 | Frithsen et al. [S34] |
| Sedentariate polychaetes | 7.25 | Ricciardi and Bourget [S35], Clarke [S36], Cauffope and Heymans [S38], Brey [S32] |
| *Mediomastus ambiseta* | 4.76 | Frithsen et al. [S34] |
| *Streblospio benedicti* | 4.8 | Frithsen et al. [S34] |
| *Polydora ligni* | 7.53 | Frithsen et al. [S34] |
| *Chaetozone sp.* | 10.76 | Frithsen et al. [S34] |
| *Ampharetidae* | 6.54 | Frithsen et al. [S34] |
| Polyplacophora | 13.6 | Ricciardi and Bourget [S35], Clarke [S36] |
| Pycnogonida | 10.4 | Galeron et al. 2000 |
| Pogonophora | 5.1 | Rowe [S39] |
| Porifera | 3.75 | Brey [S32] |
| Priapula | 3.25 | Ricciardi and Bourget [S35], Clarke [S36], Brey [S32] |
| Scaphopoda | 4 | Rowe [S39] |
| Sipuncula | 5.2 | Rowe [S39], Brey [S32] |
| Urochordata | 1.5 | Galeron et al. [S31], Brey [S32] |
| **Permanent meiofauna** |  |  |
| Foraminifera | 2 | Rudnick et al. [S40] |
| Harpacticoida | 8 | Rudnick et al. [S40] |
| Nematoda | 9 | Rudnick et al. [S40] |
