# Supplementary material for: Towards Predicting Basin-Wide Invertebrate Organic Biomass and Production in Marine Sediments from a Coastal Sea
Source: PLoS One. 2012 Jul 6;7(7):e40295. doi: 10.1371/journal.pone.0040295 (PMC3391270; doi:10.1371/journal.pone.0040295)
Supplement: Table S4 — Listing of meiofaunal biomass and production values, as shown in Figure 4 . General locations for samples are listed (zone) and cross-referenced to Figure S1. (DOC) [file pone.0040295.s005.doc]

Supporting Table S4.

| zone | year | Depth (m) | % sand | Modified organic flux (kj/0.1m^2^/yr) | Macro (>1mm) production (kj/0.1m^2^/yr) | Meio (<1mm) production (kj/0.1m^2^/yr) | Meio (<1mm) % of total production | Macro (>1mm) organic biomass (kj/0.1m^2^) | Meio (>1mm) organic biomass (kj/0.1m^2^) | Meio (<1mm) % of total biomass |
| --- | --- | --- | --- | --- | --- | --- | --- | --- | --- | --- |
| D | 2009 | 80 | 72.5 | 0.86 | 31.38 | 13.67 | 30.35% | 34.35 | 8.42 | 19.69% |
| C | 2010 | 7 | 45.0 | 2.28 | 11.60 | 5.19 | 30.91% | 8.04 | 1.78 | 18.15% |
| C | 2001 | 10 | 58.4 | 0.89 | 10.74 | 4.01 | 27.18% | 7.62 | 1.54 | 16.85% |
| C | 2000 | 76 | 59.8 | 1.22 | 18.23 | 8.27 | 31.21% | 19.48 | 3.91 | 16.73% |
| C | 2000 | 70 | 53.4 | 1.26 | 20.02 | 9.60 | 32.40% | 23.53 | 3.99 | 14.48% |
| C | 2000 | 85 | 53.9 | 1.31 | 18.65 | 12.13 | 39.41% | 19.90 | 3.13 | 13.60% |
| E | 2008 | 41 | 51.1 | 0.94 | 22.44 | 5.22 | 18.87% | 20.17 | 3.00 | 12.95% |
| C | 2000 | 70 | 65.8 | 1.13 | 17.05 | 9.45 | 35.67% | 19.46 | 2.77 | 12.48% |
| E | 2009 | 30 | 24.5 | 0.73 | 22.23 | 4.69 | 17.43% | 22.26 | 2.93 | 11.62% |
| D | 2009 | 80 | 49.0 | 1.40 | 35.25 | 10.25 | 22.53% | 36.95 | 4.73 | 11.34% |
| E | 2009 | 84 | 17.0 | 1.68 | 38.77 | 7.14 | 15.54% | 41.38 | 4.30 | 9.42% |
| C | 2001 | 10 | 62.9 | 0.89 | 11.82 | 4.26 | 26.52% | 12.33 | 1.20 | 8.87% |
| C | 2010 | 6 | 20.0 | 2.14 | 9.16 | 1.22 | 11.79% | 6.31 | 0.58 | 8.47% |
| E | 2009 | 47 | 16.0 | 1.31 | 26.00 | 5.79 | 18.20% | 30.34 | 2.70 | 8.18% |
| C | 2010 | 12 | 60.0 | 1.89 | 9.62 | 1.59 | 14.19% | 6.71 | 0.59 | 8.05% |
| D | 2008 | 80 | 49.2 | 1.43 | 43.13 | 7.74 | 15.21% | 46.14 | 3.96 | 7.91% |
| C | 2000 | 77 | 62.3 | 1.27 | 30.44 | 8.93 | 22.68% | 36.33 | 2.73 | 7.00% |
| D | 2008 | 80 | 27.6 | 4.27 | 32.61 | 6.66 | 16.96% | 56.61 | 4.03 | 6.65% |
| E | 2009 | 34 | 6.8 | 1.24 | 23.17 | 4.32 | 15.73% | 28.47 | 1.95 | 6.41% |
| E | 2009 | 65 | 3.3 | 0.92 | 28.49 | 4.68 | 14.11% | 34.11 | 2.29 | 6.28% |
| E | 2009 | 41 | 36.9 | 0.71 | 25.17 | 2.88 | 10.26% | 26.99 | 1.69 | 5.88% |
| E | 2009 | 62 | 9.8 | 1.28 | 34.25 | 4.11 | 10.73% | 35.55 | 2.19 | 5.80% |
| E | 2008 | 32 | 29.0 | 1.11 | 31.83 | 4.73 | 12.95% | 34.38 | 1.64 | 4.56% |
| D | 2008 | 80 | 21.7 | 4.17 | 35.99 | 6.62 | 15.53% | 61.83 | 2.92 | 4.50% |
| D | 2009 | 80 | 33.4 | 13.50 | 56.79 | 8.89 | 13.53% | 92.97 | 4.38 | 4.49% |
| D | 2009 | 43 | 16.5 | 1.26 | 27.85 | 2.26 | 7.49% | 31.47 | 1.39 | 4.23% |
| D | 2008 | 80 | 31.0 | 13.35 | 42.08 | 5.06 | 10.73% | 80.40 | **3.45** | 4.12% |
| C | 2000 | 80 | 59.4 | 1.16 | 29.75 | 7.04 | 19.14% | 49.56 | 2.11 | 4.09% |
| C | 2000 | 75 | 56.9 | 1.21 | 31.00 | 5.10 | 14.12% | 36.72 | 1.52 | 3.97% |
| D | 2009 | 80 | 27.4 | 3.65 | 38.72 | 8.31 | 17.67% | 104.64 | 4.13 | 3.80% |
| B | 2010 | 160 | 34.0 | 0.87 | 12.74 | 0.96 | 6.98% | 14.46 | 0.55 | 3.68% |
| E | 2009 | 54 | 6.9 | 1.21 | 31.92 | 2.67 | 7.71% | 37.62 | 1.44 | 3.68% |
| E | 2008 | 62 | 10.0 | 1.76 | 37.93 | 3.73 | 8.94% | 40.13 | 1.49 | 3.57% |
| D | 2008 | 80 | 19.4 | 2.17 | 34.27 | 2.54 | 6.90% | 41.48 | 1.52 | 3.53% |
| C | 2010 | 7 | 30.0 | 2.31 | 25.30 | 3.28 | 11.49% | 29.67 | 1.08 | 3.52% |
| E | 2008 | 54 | 6.3 | 1.47 | 26.18 | 2.38 | 8.32% | 27.44 | 0.98 | 3.45% |
| D | 2009 | 80 | 23.3 | 10.19 | 35.12 | 7.57 | 17.74% | 102.40 | 3.54 | 3.35% |
| E | 2009 | 75 | 1.8 | 0.86 | 22.67 | 1.42 | 5.89% | 26.90 | 0.91 | 3.28% |
| E | 2009 | 30 | 28.5 | 1.14 | 25.08 | 1.56 | 5.85% | 26.87 | 0.90 | 3.25% |
| B | 2010 | 245 | 21.7 | 0.54 | 2.09 | 0.19 | 8.38% | 3.64 | 0.12 | 3.20% |
| E | 2009 | 32 | 8.1 | 1.52 | 29.82 | 2.23 | 6.96% | 46.40 | 1.52 | 3.17% |
| E | 2009 | 58 | 4.1 | 0.86 | 26.67 | 2.27 | 7.84% | 33.82 | 1.10 | 3.15% |
| E | 2008 | 75 | 5.4 | 0.97 | 31.18 | 2.93 | 8.60% | 47.97 | 1.42 | 2.88% |
| E | 2008 | 47 | 29.5 | 1.59 | 38.86 | 4.72 | 10.83% | 50.79 | 1.36 | 2.61% |
| C | 2008 | 80 | 23.8 | 10.19 | 32.05 | 3.10 | 8.82% | 62.60 | 1.59 | 2.47% |
| B | 2007 | 365 | 3.0 | 0.58 | 6.59 | 0.67 | 9.23% | 13.21 | 0.33 | 2.44% |
| D | 2009 | 80 | 26.7 | 3.47 | 43.36 | 10.04 | 18.79% | 148.75 | 3.68 | 2.41% |
| E | 2008 | 58 | 10.2 | 0.86 | 34.11 | 3.24 | 8.68% | 39.32 | 0.95 | 2.36% |
| B | 2010 | 161 | 13.4 | 1.21 | 13.73 | 1.41 | 9.30% | 36.36 | 0.77 | 2.07% |
| B | 2007 | 309 | 5.0 | 0.27 | 5.68 | 0.53 | 8.54% | 17.09 | 0.36 | 2.06% |
| E | 2009 | 84 | 11.2 | 1.00 | 29.56 | 2.96 | 9.09% | 57.71 | 1.20 | 2.03% |
| E | 2008 | 84 | 3.4 | 3.00 | 45.60 | 2.06 | 4.32% | 47.62 | 0.97 | 2.00% |
| E | 2008 | 30 | 11.7 | 0.81 | 30.58 | 2.18 | 6.66% | 34.06 | 0.70 | 2.00% |
| B | 2010 | 250 | 13.2 | 0.63 | 2.88 | 0.13 | 4.35% | 4.17 | 0.08 | 1.93% |
| B | 2010 | 300 | 17.0 | 0.91 | 5.42 | 0.29 | 5.11% | 9.93 | 0.18 | 1.75% |
| E | 2008 | 34 | 8.0 | 1.16 | 26.33 | 1.42 | 5.13% | 30.10 | 0.52 | 1.70% |
| C | 2010 | 300 | 46.6 | 0.67 | 12.00 | 1.24 | 9.33% | 44.80 | 0.77 | 1.69% |
| D | 2009 | 80 | 11.6 | 4.94 | 34.15 | 4.68 | 12.05% | 109.37 | 1.88 | 1.69% |
| D | 2009 | 80 | 7.6 | 6.41 | 33.14 | 2.62 | 7.33% | 88.12 | 1.41 | 1.58% |
| E | 2008 | 43 | 9.2 | 1.61 | 36.77 | 1.54 | 4.01% | 43.08 | 0.65 | 1.49% |
| E | 2008 | 30 | 19.4 | 0.76 | 33.51 | 2.01 | 5.65% | 33.55 | 0.50 | 1.47% |
| E | 2008 | 65 | 12.3 | 0.75 | 38.37 | 2.10 | 5.18% | 55.83 | 0.68 | 1.20% |
| E | 2008 | 84 | 2.8 | 0.82 | 30.15 | 1.73 | 5.43% | 78.08 | 0.65 | 0.83% |
