# Supplementary material for: Towards Predicting Basin-Wide Invertebrate Organic Biomass and Production in Marine Sediments from a Coastal Sea
Source: PLoS One. 2012 Jul 6;7(7):e40295. doi: 10.1371/journal.pone.0040295 (PMC3391270; doi:10.1371/journal.pone.0040295)
Supplement: Table S5 — Summary (means for all sample locations and time) organic biomass and production measurements for macrofaunal (>1 mm) organisms, along with estimates (from the exponential regression with %sand) for small faunal (<1 mm) for each location – Estimated totals are combined for all invertebrate organisms, as shown in Figs. 5 , 6 , 7 . Environmental variables are also included. Note that modified organic flux values were not available for all samples. The general sampling zone is cross-referenced to Figure S1. (DOC) [file pone.0040295.s006.doc]

Supporting Table S5.

| Sample zone | year | Depth (m) | %sand | Organic flux/ del 15N (kj/0.1m^2^/yr) | Predicted biomass for fauna <0.1mm (kj/0.1m^2^) | Macrofaunal (>1mm) biomass (kj/0.1m^2^) | Estimated total biomass (kj/0.1m^2^) | Predicted production for fauna <0.1 mm (kj/0.1m^2^/yr) | Macrofaunal (>1mm) production (kj/0.1m^2^/yr) | Estimated total production (kj/0.1m^2^/yr) | Total Production/ Biomass |
| --- | --- | --- | --- | --- | --- | --- | --- | --- | --- | --- | --- |
|  |  |  |  |  |  |  |  |  |  |  |  |
| B | 2003 | 19 | 70 | 0.67 | 5.16 | 43.88 | 49.03 | 11.72 | 31.55 | 43.27 | 0.88 |
| B | 2003 | 19 | 32 | 0.79 | 1.13 | 23.09 | 24.22 | 3.10 | 20.00 | 23.09 | 0.95 |
| B | 2010 | 300 | 46.62 | 0.55 | 3.07 | 44.80 | 47.87 | 2.60 | 12.00 | 14.60 | 0.31 |
| B | 2010 | 161 | 13.37 | 1.16 | 1.16 | 36.36 | 37.52 | 1.38 | 13.73 | 15.11 | 0.40 |
| B | 2010 | 300 | 16.97 | 0.63 | 0.34 | 9.93 | 10.27 | 0.59 | 5.42 | 6.02 | 0.59 |
| B | 2010 | 160 | 34 | 0.21 | 0.51 | 9.87 | 10.37 | 1.76 | 10.85 | 12.61 | 1.22 |
| B | 2010 | 250 | 13.2 | 0.54 | 0.13 | 4.17 | 4.31 | 0.29 | 2.88 | 3.17 | 0.74 |
| B | 2010 | 245 | 21.7 | 0.49 | 0.14 | 3.64 | 3.78 | 0.26 | 2.09 | 2.35 | 0.62 |
| D | 1991 | 121 | 78.1 | 2.16 | 6.08 | 42.94 | 49.02 | 13.67 | 30.52 | 44.19 | 0.90 |
| D | 1997 | 19 | 79.28 |  | 5.76 | 39.60 | 45.36 | 18.41 | 40.03 | 58.44 | 1.29 |
| D | 1992 | 23 | 5.9 |  | 0.96 | 35.92 | 36.89 | 2.42 | 28.50 | 30.92 | 0.84 |
| D | 1990 | 23 | 3 |  | 0.79 | 31.61 | 32.41 | 1.81 | 22.81 | 24.62 | 0.76 |
| D | 2002 | 19 | 5 |  | 0.80 | 30.55 | 31.36 | 2.59 | 31.12 | 33.71 | 1.08 |
| D | 1991 | 117 | 75.7 | 0.76 | 3.90 | 29.15 | 33.05 | 9.25 | 21.83 | 31.07 | 0.94 |
| D | 1994 | 123 | 77 | 2.55 | 3.44 | 24.90 | 28.34 | 9.10 | 20.85 | 29.95 | 1.06 |
| D | 1993 | 23 | 6 |  | 0.57 | 21.32 | 21.89 | 1.62 | 18.99 | 20.61 | 0.94 |
| D | 1994 | 118 | 64 | 0.83 | 2.13 | 20.77 | 22.89 | 5.19 | 16.03 | 21.21 | 0.93 |
| D | 1989 | 23 | 6.68 |  | 0.57 | 20.76 | 21.33 | 2.91 | 33.69 | 36.60 | 1.72 |
| D | 1994 | 24 | 5 |  | 0.52 | 19.61 | 20.12 | 1.37 | 16.42 | 17.78 | 0.88 |
| D | 1997 | 4 | 47.51 |  | 1.03 | 14.78 | 15.82 | 2.56 | 11.56 | 14.12 | 0.89 |
| D | 1991 | 23 | 4.2 |  | 0.36 | 14.00 | 14.36 | 1.13 | 13.85 | 14.98 | 1.04 |
| C | 2008 | 25 | 97 | 0.64 | 8.64 | 39.48 | 48.12 | 27.88 | 40.29 | 68.17 | 1.42 |
| C | 2004 | 25 | 80 | 0.75 | 4.82 | 32.60 | 37.42 | 9.04 | 19.32 | 28.35 | 0.76 |
| C | 2010 | 7 | 30 | 0.81 | 1.39 | 29.67 | 31.06 | 3.74 | 25.30 | 29.04 | 0.93 |
| C | 2010 | 5 | 10 | 1.03 | 0.26 | 8.73 | 8.99 | 1.05 | 11.30 | 12.35 | 1.37 |
| C | 2010 | 7 | 45 | 0.53 | 0.53 | 8.04 | 8.57 | 2.42 | 11.60 | 14.03 | 1.64 |
| C | 2010 | 12 | 60 | 0.53 | 0.63 | 6.71 | 7.33 | 2.84 | 9.62 | 12.46 | 1.70 |
| C | 2010 | 6 | 20 | 0.51 | 0.23 | 6.31 | 6.54 | 1.08 | 9.16 | 10.24 | 1.56 |
| D | 2000 | 223 | 26.1033 | 1.32 | 2.21 | 51.78 | 53.99 | 4.76 | 35.23 | 39.99 | 0.74 |
| D | 1990 | 223 | 55 | 0.71 | 3.36 | 40.38 | 43.74 | 7.67 | 29.15 | 36.82 | 0.84 |
| D | 2005 | 223 | 22.8 | 1.31 | 1.09 | 27.42 | 28.51 | 3.10 | 24.78 | 27.88 | 0.98 |
| D | 2006 | 223 | 35.8667 | 0.92 | 1.29 | 24.19 | 25.48 | 3.55 | 20.96 | 24.50 | 0.96 |
| D | 2001 | 223 | 21.2 | 1.48 | 0.90 | 23.61 | 24.52 | 1.91 | 15.81 | 17.72 | 0.72 |
| D | 1989 | 223 | 67.35 | 1.05 | 2.25 | 20.37 | 22.63 | 6.27 | 17.93 | 24.20 | 1.07 |
| D | 2002 | 223 | 36.13 | 1.15 | 1.04 | 19.33 | 20.37 | 2.14 | 12.58 | 14.72 | 0.72 |
| D | 1991 | 223 | 36.3 | 1.13 | 0.82 | 15.15 | 15.97 | 1.92 | 11.24 | 13.16 | 0.82 |
| D | 2007 | 223 | 30.9 | 1.48 | 0.67 | 14.12 | 14.79 | 2.05 | 13.60 | 15.65 | 1.06 |
| D | 1997 | 223 | 14.12 | 1.54 | 0.43 | 13.15 | 13.58 | 0.85 | 8.30 | 9.15 | 0.67 |
| D | 2003 | 223 | 11.7245 | 1.63 | 0.32 | 10.37 | 10.69 | 0.85 | 8.73 | 9.57 | 0.90 |
| D | 2004 | 223 | 35.2 | 1.32 | 0.50 | 9.55 | 10.06 | 1.36 | 8.17 | 9.53 | 0.95 |
| D | 1995 | 223 | 25 | 1.22 | 0.34 | 8.23 | 8.58 | 0.88 | 6.68 | 7.56 | 0.88 |
| D | 1994 | 223 | 37 | 1.08 | 0.43 | 7.90 | 8.34 | 1.01 | 5.78 | 6.79 | 0.81 |
| D | 1998 | 223 | 22.54 | 0.34 | 0.20 | 5.16 | 5.36 | 0.50 | 4.02 | 4.53 | 0.84 |
| D | 1993 | 223 | 41 | 0.94 | 0.29 | 4.80 | 5.09 | 0.93 | 4.88 | 5.81 | 1.14 |
| D | 1999 | 223 | 22.69 | 0.34 | 0.15 | 3.69 | 3.83 | 0.34 | 2.69 | 3.03 | 0.79 |
| B | 2001 | 34 | 94.7 | 0.47 | 8.05 | 38.78 | 46.83 | 21.65 | 33.00 | 54.65 | 1.17 |
| B | 2001 | 30 | 95.3 | 0.74 | 3.69 | 17.56 | 21.25 | 10.60 | 15.93 | 26.52 | 1.25 |
| B | 2001 | 32 | 98.8 | 0.51 | 3.30 | 14.45 | 17.75 | 8.07 | 11.20 | 19.27 | 1.09 |
| B | 2000 | 52 | 37.18 | 0.40 | 0.86 | 15.53 | 16.39 | 2.63 | 15.06 | 17.69 | 1.08 |
| B | 2005 | 65 | 66 | 1.70 | 6.50 | 60.63 | 67.13 | 19.99 | 59.00 | 78.99 | 1.18 |
| B | 2005 | 70 | 86 | 0.65 | 8.54 | 50.31 | 58.85 | 27.75 | 51.67 | 79.42 | 1.35 |
| B | 2005 | 65 | 85 | 1.84 | 8.33 | 50.18 | 58.51 | 24.08 | 45.89 | 69.97 | 1.20 |
| B | 2005 | 70 | 74 | 1.90 | 5.11 | 39.66 | 44.77 | 15.87 | 38.96 | 54.83 | 1.22 |
| B | 2005 | 75 | 50 | 2.93 | 2.40 | 32.42 | 34.83 | 7.92 | 33.77 | 41.69 | 1.20 |
| B | 2005 | 65 | 77 | 1.52 | 3.94 | 28.54 | 32.48 | 13.49 | 30.89 | 44.38 | 1.37 |
| B | 2005 | 65 | 60 | 0.85 | 1.96 | 21.03 | 22.99 | 6.37 | 21.58 | 27.95 | 1.22 |
| B | 2005 | 60 | 99 | 0.83 | 4.62 | 20.15 | 24.76 | 16.84 | 23.25 | 40.09 | 1.62 |
| B | 2005 | 60 | 92 | 0.80 | 2.01 | 10.30 | 12.31 | 7.70 | 12.49 | 20.19 | 1.64 |
| C | 2001 | 25 | 82.67 | 0.53 | 4.73 | 30.05 | 34.77 | 12.41 | 24.95 | 37.37 | 1.07 |
| C | 2001 | 25 | 83 | 0.53 | 4.05 | 25.55 | 29.60 | 11.10 | 22.15 | 33.26 | 1.12 |
| C | 2001 | 18 | 79.99 | 0.59 | 2.90 | 19.64 | 22.54 | 7.94 | 16.97 | 24.91 | 1.10 |
| C | 2001 | 25 | 83 | 0.53 | 2.98 | 18.78 | 21.76 | 5.01 | 9.99 | 14.99 | 0.69 |
| C | 2001 | 25 | 82.79 | 0.53 | 2.96 | 18.78 | 21.74 | 7.95 | 15.94 | 23.89 | 1.10 |
| C | 2001 | 18 | 83 | 0.59 | 2.64 | 16.63 | 19.27 | 7.38 | 14.73 | 22.11 | 1.15 |
| C | 2001 | 25 | 84.42 | 0.54 | 2.63 | 16.06 | 18.69 | 8.24 | 15.92 | 24.16 | 1.29 |
| C | 2001 | 18 | 85.46 | 0.59 | 2.03 | 12.10 | 14.13 | 6.74 | 12.71 | 19.44 | 1.38 |
| C | 2001 | 5 | 92.02 | 0.27 | 2.23 | 11.43 | 13.66 | 7.76 | 12.57 | 20.33 | 1.49 |
| C | 2001 | 18 | 84.77 | 0.59 | 1.73 | 10.48 | 12.21 | 5.84 | 11.18 | 17.02 | 1.39 |
| C | 2001 | 10 | 90.21 | 0.36 | 1.86 | 9.95 | 11.82 | 2.95 | 4.99 | 7.95 | 0.67 |
| C | 2001 | 18 | 80 | 0.58 | 1.13 | 7.63 | 8.76 | 4.35 | 9.30 | 13.64 | 1.56 |
| C | 2001 | 5 | 95.76 | 0.26 | 1.28 | 6.01 | 7.29 | 5.42 | 8.06 | 13.49 | 1.85 |
| C | 2001 | 10 | 93 | 0.35 | 0.80 | 4.02 | 4.82 | 3.17 | 5.03 | 8.20 | 1.70 |
| C | 2001 | 5 | 97 | 0.26 | 0.84 | 3.83 | 4.67 | 4.27 | 6.17 | 10.44 | 2.24 |
| C | 2001 | 5 | 97 | 0.26 | 0.76 | 3.49 | 4.25 | 3.88 | 5.61 | 9.49 | 2.23 |
| C | 2001 | 10 | 93.52 | 0.35 | 0.70 | 3.46 | 4.16 | 3.07 | 4.81 | 7.89 | 1.89 |
| C | 2001 | 10 | 93 | 0.35 | 0.47 | 2.34 | 2.81 | 1.94 | 3.07 | 5.01 | 1.78 |
| C | 2001 | 10 | 93.29 | 0.35 | 0.42 | 2.10 | 2.52 | 2.24 | 3.53 | 5.78 | 2.29 |
| C | 2001 | 5 | 95.76 | 0.26 | 0.31 | 1.46 | 1.77 | 1.63 | 2.42 | 4.04 | 2.29 |
| E | 2004 | 34 | 5 | 1.18 | 2.26 | 85.95 | 88.21 | 3.36 | 40.35 | 43.71 | 0.50 |
| E | 2003 | 47 | 23.2 | 1.29 | 3.24 | 80.90 | 84.14 | 5.56 | 43.98 | 49.54 | 0.59 |
| E | 2008 | 84 | 2.8 | 0.82 | 1.95 | 78.08 | 80.04 | 2.38 | 30.15 | 32.54 | 0.41 |
| E | 2006 | 84 | 10.8 | 1.54 | 2.16 | 71.79 | 73.95 | 3.59 | 37.73 | 41.31 | 0.56 |
| E | 2004 | 84 | 1.7 | 0.91 | 1.65 | 67.75 | 69.40 | 2.52 | 32.64 | 35.15 | 0.51 |
| E | 2005 | 58 | 5.3 | 0.86 | 1.78 | 67.09 | 68.87 | 3.16 | 37.72 | 40.88 | 0.59 |
| E | 2004 | 47 | 18.8 | 1.20 | 2.11 | 58.42 | 60.53 | 4.99 | 43.66 | 48.65 | 0.80 |
| E | 2009 | 84 | 11.2 | 1.00 | 1.75 | 57.71 | 59.46 | 2.84 | 29.56 | 32.39 | 0.54 |
| E | 2004 | 58 | 3.9 | 0.86 | 1.45 | 56.56 | 58.01 | 3.24 | 39.96 | 43.21 | 0.74 |
| E | 2004 | 65 | 2.8 | 0.97 | 1.41 | 56.54 | 57.95 | 3.13 |  | 3.13 | 0.05 |
| E | 2008 | 65 | 12.3 | 0.75 | 1.74 | 55.83 | 57.57 | 3.78 | 38.37 | 42.15 | 0.73 |
| E | 2005 | 75 | 1.8 | 0.81 | 1.32 | 54.03 | 55.35 | 2.09 | 27.09 | 29.18 | 0.53 |
| E | 2005 | 19 | 25.3 | 1.24 | 2.25 | 53.57 | 55.82 | 5.20 | 39.14 | 44.34 | 0.79 |
| E | 1995 | 54 | 7 | 0.73 | 1.46 | 53.02 | 54.48 | 3.92 | 44.99 | 48.90 | 0.90 |
| E | 2002 | 34 | 9.5 | 1.39 | 1.54 | 52.78 | 54.32 | 3.20 | 34.73 | 37.93 | 0.70 |
| E | 2003 | 43 | 18.1 | 1.30 | 1.87 | 52.50 | 54.36 | 4.46 | 39.67 | 44.13 | 0.81 |
| E | 2005 | 34 | 8.1 | 1.20 | 1.48 | 52.30 | 53.77 | 3.44 | 38.47 | 41.91 | 0.78 |
| E | 2005 | 84 | 4 | 0.91 | 1.33 | 51.64 | 52.97 | 2.28 | 28.09 | 30.38 | 0.57 |
| E | 2005 | 43 | 18.7 | 1.30 | 1.83 | 50.89 | 52.72 | 4.57 | 40.04 | 44.60 | 0.85 |
| E | 2008 | 47 | 29.5 | 1.59 | 2.35 | 50.79 | 53.14 | 5.68 | 38.86 | 44.55 | 0.84 |
| E | 2003 | 34 | 5.4 | 1.14 | 1.34 | 50.65 | 51.99 | 3.29 | 39.21 | 42.50 | 0.82 |
| E | 2003 | 65 | 2.8 | 0.90 | 1.23 | 49.03 | 50.26 | 2.91 | 36.83 | 39.74 | 0.79 |
| E | 2008 | 75 | 5.4 | 0.97 | 1.27 | 47.97 | 49.24 | 2.62 | 31.18 | 33.79 | 0.69 |
| E | 2004 | 54 | 6.1 | 1.11 | 1.29 | 47.89 | 49.18 | 3.27 | 38.35 | 41.62 | 0.85 |
| E | 2005 | 65 | 2.7 | 0.92 | 1.19 | 47.81 | 49.01 | 2.85 | 36.09 | 38.94 | 0.79 |
| E | 2008 | 84 | 3.4 | 3.00 | 1.21 | 47.62 | 48.83 | 3.66 | 45.60 | 49.25 | 1.01 |
| E | 2003 | 75 | 1.8 | 0.86 | 1.16 | 47.60 | 48.76 | 2.72 | 35.22 | 37.94 | 0.78 |
| E | 2002 | 47 | 19.3 | 1.43 | 1.73 | 47.44 | 49.18 | 4.54 | 39.26 | 43.80 | 0.89 |
| E | 2004 | 43 | 17.9 | 1.48 | 1.62 | 45.89 | 47.52 | 4.02 | 35.95 | 39.97 | 0.84 |
| E | 2007 | 60 | 5.2 | 1.13 | 1.19 | 45.21 | 46.40 | 2.80 | 33.46 | 36.25 | 0.78 |
| E | 2006 | 65 | 3.5 | 0.85 | 1.14 | 44.77 | 45.90 | 2.62 | 32.59 | 35.20 | 0.77 |
| E | 2007 | 44 | 18.9 | 1.19 | 1.62 | 44.74 | 46.36 | 3.83 | 33.42 | 37.25 | 0.80 |
| E | 2003 | 58 | 3.7 | 0.86 | 1.13 | 44.23 | 45.36 | 2.76 | 34.15 | 36.91 | 0.81 |
| E | 2007 | 46 | 11.3 | 1.02 | 1.32 | 43.51 | 44.83 | 3.51 | 36.48 | 39.99 | 0.89 |
| E | 2008 | 43 | 9.2 | 1.61 | 1.25 | 43.08 | 44.32 | 3.37 | 36.77 | 40.14 | 0.91 |
| E | 2004 | 75 | 1.4 | 0.85 | 1.04 | 42.83 | 43.87 | 2.14 | 27.91 | 30.05 | 0.69 |
| E | 2006 | 84 | 2.9 | 0.87 | 1.07 | 42.76 | 43.83 | 1.87 | 23.64 | 25.52 | 0.58 |
| E | 2002 | 43 | 19.2 | 1.48 | 1.54 | 42.10 | 43.63 | 3.82 | 33.10 | 36.92 | 0.85 |
| E | 2006 | 75 | 2.1 | 0.74 | 1.03 | 41.80 | 42.82 | 2.23 | 28.61 | 30.84 | 0.72 |
| E | 2009 | 84 | 2.3 | 1.68 | 1.02 | 41.38 | 42.40 | 3.03 | 38.77 | 41.80 | 0.99 |
| E | 2007 | 73 | 1.8 | 0.71 | 1.00 | 40.86 | 41.86 | 2.39 | 30.90 | 33.29 | 0.80 |
| E | 2006 | 62 | 11 | 1.19 | 1.22 | 40.45 | 41.67 | 3.38 | 35.35 | 38.73 | 0.93 |
| E | 2008 | 62 | 1.8 | 1.76 | 0.98 | 40.13 | 41.11 | 2.93 | 37.93 | 40.86 | 0.99 |
| E | 2007 | 81 | 10.8 | 1.30 | 1.20 | 39.94 | 41.14 | 3.59 | 37.78 | 41.37 | 1.01 |
| E | 2008 | 58 | 10.2 | 0.86 | 1.17 | 39.32 | 40.49 | 3.20 | 34.11 | 37.31 | 0.92 |
| E | 2007 | 81 | 2.1 | 0.89 | 0.96 | 39.11 | 40.07 | 2.41 | 30.99 | 33.40 | 0.83 |
| E | 2009 | 54 | 6.9 | 1.21 | 1.03 | 37.62 | 38.65 | 2.77 | 31.92 | 34.69 | 0.90 |
| E | 2003 | 54 | 5.7 | 1.06 | 1.00 | 37.44 | 38.44 | 2.67 | 31.55 | 34.21 | 0.89 |
| E | 1995 | 62 | 5 | 0.79 | 0.98 | 37.38 | 38.36 | 2.83 | 33.97 | 36.79 | 0.96 |
| E | 2006 | 58 | 5.7 | 0.86 | 1.00 | 37.31 | 38.31 | 2.32 | 27.50 | 29.82 | 0.78 |
| E | 2007 | 55 | 14.7 | 0.86 | 1.21 | 36.82 | 38.03 | 3.18 | 30.58 | 33.76 | 0.89 |
| E | 2009 | 62 | 9.8 | 1.28 | 1.04 | 35.55 | 36.60 | 3.18 | 34.25 | 37.43 | 1.02 |
| E | 2007 | 59 | 3.2 | 1.32 | 0.88 | 34.73 | 35.60 | 2.61 | 32.75 | 35.36 | 0.99 |
| E | 2007 | 51 | 7 | 1.01 | 0.94 | 34.17 | 35.11 | 2.59 | 29.79 | 32.39 | 0.92 |
| E | 2009 | 65 | 3.3 | 0.92 | 0.86 | 34.11 | 34.97 | 2.28 | 28.49 | 30.77 | 0.88 |
| E | 2005 | 54 | 8.4 | 1.17 | 0.96 | 33.87 | 34.83 | 2.64 | 29.34 | 31.98 | 0.92 |
| E | 2009 | 58 | 4.1 | 0.86 | 0.87 | 33.82 | 34.69 | 2.17 | 26.67 | 28.85 | 0.83 |
| E | 2006 | 47 | 19.1 | 1.19 | 1.22 | 33.56 | 34.78 | 3.08 | 26.75 | 29.83 | 0.86 |
| E | 2008 | 30 | 19.4 | 0.76 | 1.23 | 33.55 | 34.78 | 3.88 | 33.51 | 37.39 | 1.07 |
| E | 2007 | 52 | 28.4 | 0.64 | 1.50 | 33.33 | 34.83 | 4.50 | 31.56 | 36.06 | 1.04 |
| E | 2002 | 75 | 1.3 | 0.76 | 0.80 | 33.12 | 33.92 | 1.94 | 25.39 | 27.32 | 0.81 |
| E | 2005 | 47 | 19.5 | 1.37 | 1.21 | 32.95 | 34.16 | 3.37 | 28.99 | 32.35 | 0.95 |
| E | 2002 | 58 | 2.7 | 0.86 | 0.82 | 32.87 | 33.69 | 2.25 | 28.51 | 30.76 | 0.91 |
| E | 2002 | 84 | 2.1 | 0.77 | 0.78 | 31.76 | 32.54 | 1.99 | 25.60 | 27.59 | 0.85 |
| E | 2009 | 43 | 16.5 | 1.26 | 1.08 | 31.47 | 32.55 | 3.02 | 27.85 | 30.87 | 0.95 |
| E | 2003 | 84 | 1.8 | 0.91 | 0.76 | 31.26 | 32.03 | 1.98 | 25.66 | 27.65 | 0.86 |
| E | 2006 | 54 | 5.8 | 0.99 | 0.82 | 30.44 | 31.26 | 2.20 | 26.01 | 28.22 | 0.90 |
| E | 2009 | 47 | 13.5 | 1.31 | 0.97 | 30.34 | 31.31 | 2.63 | 26.00 | 28.63 | 0.91 |
| E | 2008 | 34 | 8 | 1.16 | 0.85 | 30.10 | 30.95 | 2.35 | 26.33 | 28.67 | 0.93 |
| E | 2006 | 43 | 17.5 | 1.24 | 1.04 | 29.65 | 30.69 | 2.68 | 24.17 | 26.85 | 0.87 |
| E | 2002 | 65 | 2.7 | 0.93 | 0.72 | 28.86 | 29.58 | 1.79 | 22.69 | 24.48 | 0.83 |
| E | 2009 | 34 | 6.8 | 1.24 | 0.78 | 28.47 | 29.25 | 2.01 | 23.17 | 25.17 | 0.86 |
| E | 2007 | 32 | 5.9 | 1.26 | 0.76 | 28.22 | 28.98 | 2.13 | 25.03 | 27.15 | 0.94 |
| E | 2008 | 54 | 6.3 | 1.47 | 0.74 | 27.44 | 28.18 | 2.24 | 26.18 | 28.42 | 1.01 |
| E | 2009 | 41 | 36.9 | 0.71 | 1.48 | 26.99 | 28.47 | 4.36 | 25.17 | 29.54 | 1.04 |
| E | 2009 | 75 | 1.8 | 0.86 | 0.66 | 26.90 | 27.55 | 1.75 | 22.67 | 24.42 | 0.89 |
| E | 2002 | 54 | 5.8 | 0.92 | 0.71 | 26.44 | 27.14 | 2.08 | 24.60 | 26.68 | 0.98 |
| E | 2009 | 30 | 24.5 | 0.73 | 0.92 | 22.26 | 23.18 | 2.90 | 22.23 | 25.12 | 1.08 |
| E | 2006 | 34 | 5.8 | 1.17 | 0.54 | 20.33 | 20.88 | 1.42 | 16.72 | 18.14 | 0.87 |
| E | 2008 | 41 | 51.1 | 0.94 | 1.53 | 20.17 | 21.70 | 5.39 | 22.44 | 27.83 | 1.28 |
| E | 2007 | 41 | 34.5 | 0.63 | 1.00 | 19.24 | 20.24 | 3.20 | 19.49 | 22.69 | 1.12 |
| A | 2003 | 36 | 13.6 | 0.86 | 1.55 | 48.32 | 49.87 | 4.24 | 41.84 | 46.08 | 0.92 |
| A | 2003 | 52 | 84.27 | 0.81 | 1.92 | 11.77 | 13.69 | 5.27 | 10.21 | 15.48 | 1.13 |
| D | 2003 | 120 | 17 | 11.68 | 8.49 | 244.92 | 253.42 | 6.20 | 56.52 | 62.72 | 0.25 |
| D | 2003 | 80 | 16.8 | 10.19 | 7.13 | 206.53 | 213.66 | 6.10 | 55.86 | 61.96 | 0.29 |
| D | 2002 | 80 | 8.8 | 4.79 | 5.71 | 198.89 | 204.60 | 4.84 | 53.34 | 58.19 | 0.28 |
| D | 2006 | 80 | 19.9 | 10.19 | 7.15 | 192.80 | 199.95 | 5.75 | 49.09 | 54.84 | 0.27 |
| D | 2004 | 80 | 32.5 | 14.69 | 8.96 | 180.85 | 189.81 | 9.08 | 57.97 | 67.05 | 0.35 |
| D | 2003 | 80 | 4.5 | 7.59 | 4.55 | 174.91 | 179.46 | 3.28 | 39.84 | 43.12 | 0.24 |
| D | 2003 | 80 | 7.1 | 6.04 | 4.59 | 166.29 | 170.88 | 3.27 | 37.45 | 40.72 | 0.24 |
| D | 2001 | 80 | 17.1 | 16.62 | 5.57 | 160.32 | 165.89 | 6.44 | 58.64 | 65.09 | 0.39 |
| D | 2001 | 80 | 21.3 | 9.95 | 5.90 | 154.21 | 160.12 | 4.76 | 39.32 | 44.08 | 0.28 |
| D | 2002 | 80 | 6.4 | 6.29 | 4.19 | 154.06 | 158.25 | 3.46 | 40.28 | 43.74 | 0.28 |
| D | 2004 | 80 | 9.8 | 5.39 | 4.52 | 153.85 | 158.37 | 4.26 | 45.86 | 50.13 | 0.32 |
| D | 2006 | 80 | 24.7 | 3.93 | 6.20 | 149.86 | 156.07 | 6.39 | 48.79 | 55.18 | 0.35 |
| D | 2009 | 80 | 26.7 | 3.47 | 6.45 | 148.75 | 155.20 | 5.94 | 43.36 | 49.31 | 0.32 |
| D | 2003 | 100 | 17 | 13.36 | 4.97 | 143.40 | 148.38 | 4.55 | 41.50 | 46.05 | 0.31 |
| D | 2004 | 80 | 7 | 5.96 | 3.90 | 141.76 | 145.67 | 3.07 | 35.25 | 38.32 | 0.26 |
| D | 2007 | 120 | 22.1 | 7.69 | 5.28 | 135.52 | 140.81 | 4.68 | 37.96 | 42.64 | 0.30 |
| D | 2004 | 80 | 21 | 10.19 | 4.98 | 130.95 | 135.93 | 5.56 | 46.28 | 51.85 | 0.38 |
| D | 2001 | 80 | 6.5 | 7.39 | 3.51 | 128.79 | 132.30 | 3.40 | 39.48 | 42.88 | 0.32 |
| D | 2007 | 80 | 29.5 | 16.32 | 5.79 | 125.33 | 131.12 | 6.74 | 46.09 | 52.83 | 0.40 |
| D | 2002 | 80 | 15.3 | 4.40 | 4.16 | 124.81 | 128.98 | 5.79 | 54.89 | 60.68 | 0.47 |
| D | 2005 | 80 | 20.5 | 10.19 | 4.62 | 123.01 | 127.64 | 5.97 | 50.20 | 56.17 | 0.44 |
| D | 2006 | 80 | 12.7 | 4.99 | 3.72 | 118.41 | 122.13 | 3.90 | 39.27 | 43.17 | 0.35 |
| D | 2001 | 80 | 31.7 | 10.19 | 5.72 | 117.53 | 123.24 | 6.59 | 42.84 | 49.43 | 0.40 |
| D | 2005 | 80 | 11.1 | 6.04 | 3.50 | 115.55 | 119.05 | 4.04 | 42.25 | 46.29 | 0.39 |
| D | 2005 | 80 | 22 | 6.53 | 4.39 | 112.91 | 117.30 | 6.69 | 54.40 | 61.09 | 0.52 |
| D | 2009 | 80 | 11.6 | 4.94 | 3.35 | 109.37 | 112.71 | 3.31 | 34.15 | 37.45 | 0.33 |
| D | 2002 | 80 | 28.1 | 13.95 | 4.89 | 109.25 | 114.14 | 8.76 | 61.90 | 70.66 | 0.62 |
| D | 2009 | 80 | 27.4 | 3.65 | 4.61 | 104.64 | 109.25 | 5.39 | 38.72 | 44.11 | 0.40 |
| D | 2009 | 80 | 23.3 | 10.19 | 4.11 | 102.40 | 106.51 | 4.45 | 35.12 | 39.57 | 0.37 |
| D | 2007 | 80 | 26.6 | 3.70 | 4.31 | 99.61 | 103.91 | 5.74 | 41.98 | 47.72 | 0.46 |
| D | 2006 | 80 | 7.8 | 6.35 | 2.78 | 99.25 | 102.03 | 2.92 | 32.86 | 35.78 | 0.35 |
| D | 2002 | 80 | 17.7 | 10.19 | 3.34 | 94.85 | 98.19 | 5.06 | 45.43 | 50.50 | 0.51 |
| D | 2009 | 80 | 33.4 | 13.50 | 4.70 | 92.97 | 97.67 | 9.08 | 56.79 | 65.88 | 0.67 |
| D | 2005 | 80 | 7.4 | 7.25 | 2.57 | 92.42 | 94.98 | 3.35 | 38.16 | 41.51 | 0.44 |
| D | 2007 | 60 | 15.4 | 10.78 | 3.08 | 92.11 | 95.19 | 3.89 | 36.78 | 40.67 | 0.43 |
| D | 2004 | 80 | 25.8 | 5.36 | 3.86 | 90.91 | 94.77 | 6.81 | 50.74 | 57.56 | 0.61 |
| D | 2002 | 80 | 22.6 | 3.94 | 3.56 | 90.14 | 93.70 | 6.70 | 53.71 | 60.41 | 0.64 |
| D | 2006 | 80 | 28.5 | 14.84 | 4.04 | 89.43 | 93.47 | 6.37 | 44.60 | 50.97 | 0.55 |
| D | 2003 | 80 | 27.5 | 14.99 | 3.93 | 88.89 | 92.82 | 5.49 | 39.32 | 44.81 | 0.48 |
| D | 2009 | 80 | 7.6 | 6.41 | 2.46 | 88.12 | 90.58 | 2.93 | 33.14 | 36.06 | 0.40 |
| D | 2007 | 80 | 21.6 | 10.19 | 3.34 | 86.72 | 90.06 | 4.76 | 39.02 | 43.77 | 0.49 |
| D | 2001 | 80 | 14.3 | 5.17 | 2.80 | 85.88 | 88.68 | 5.58 | 54.12 | 59.70 | 0.67 |
| D | 2007 | 80 | 10.7 | 4.99 | 2.54 | 84.53 | 87.07 | 3.39 | 35.76 | 39.15 | 0.45 |
| D | 2003 | 80 | 11.6 | 4.88 | 2.52 | 82.39 | 84.92 | 4.43 | 45.74 | 50.17 | 0.59 |
| D | 2006 | 80 | 25.9 | 4.13 | 3.48 | 81.72 | 85.19 | 6.13 | 45.58 | 51.72 | 0.61 |
| D | 2008 | 80 | 31 | 13.35 | 3.85 | 80.40 | 84.25 | 6.37 | 42.08 | 48.45 | 0.58 |
| D | 2004 | 80 | 19.5 | 4.12 | 2.93 | 79.84 | 82.77 | 4.99 | 42.95 | 47.93 | 0.58 |
| D | 2007 | 80 | 8.9 | 6.18 | 2.20 | 76.49 | 78.69 | 3.02 | 33.17 | 36.19 | 0.46 |
| D | 2003 | 80 | 12.4 | 5.42 | 2.22 | 71.32 | 73.54 | 4.01 | 40.69 | 44.71 | 0.61 |
| D | 2005 | 80 | 26.2 | 16.32 | 2.89 | 67.38 | 70.27 | 7.06 | 52.13 | 59.19 | 0.84 |
| D | 2007 | 80 | 26 | 3.79 | 2.84 | 66.59 | 69.43 | 5.52 | 40.92 | 46.44 | 0.67 |
| D | 2001 | 80 | 47.2 | 2.01 | 4.43 | 63.80 | 68.23 | 11.07 | 50.39 | 61.46 | 0.90 |
| D | 2008 | 80 | 23.8 | 10.19 | 2.54 | 62.60 | 65.14 | 4.11 | 32.05 | 36.16 | 0.56 |
| D | 2008 | 80 | 21.7 | 4.17 | 2.39 | 61.83 | 64.22 | 4.40 | 35.99 | 40.39 | 0.63 |
| D | 2003 | 80 | 53 | 1.31 | 4.86 | 61.17 | 66.03 | 12.91 | 51.40 | 64.31 | 0.97 |
| D | 2002 | 80 | 64.2 | 1.20 | 6.21 | 60.39 | 66.60 | 14.95 | 46.00 | 60.95 | 0.92 |
| D | 2001 | 80 | 9.8 | 6.02 | 1.74 | 59.18 | 60.92 | 3.30 | 35.49 | 38.78 | 0.64 |
| D | 2002 | 80 | 47 | 1.74 | 4.01 | 57.90 | 61.90 | 11.09 | 50.67 | 61.76 | 1.00 |
| D | 2008 | 80 | 27.6 | 4.27 | 2.51 | 56.61 | 59.12 | 4.56 | 32.61 | 37.17 | 0.63 |
| D | 2004 | 80 | 49.4 | 1.52 | 4.12 | 56.34 | 60.46 | 10.32 | 44.64 | 54.96 | 0.91 |
| D | 2005 | 80 | 23.7 | 4.70 | 2.14 | 52.95 | 55.09 | 5.49 | 42.91 | 48.40 | 0.88 |
| D | 2007 | 80 | 51.2 | 1.25 | 3.85 | 50.47 | 54.32 | 8.78 | 36.45 | 45.24 | 0.83 |
| D | 2003 | 80 | 65.1 | 0.86 | 5.21 | 49.68 | 54.89 | 13.14 | 39.60 | 52.74 | 0.96 |
| D | 2005 | 80 | 70.5 | 1.04 | 5.87 | 49.36 | 55.23 | 15.24 | 40.55 | 55.79 | 1.01 |
| D | 2003 | 60 | 27.5 | 14.67 | 2.09 | 47.32 | 49.41 | 5.70 | 40.82 | 46.52 | 0.94 |
| D | 2008 | 80 | 49.2 | 1.43 | 3.36 | 46.14 | 49.50 | 9.93 | 43.13 | 53.06 | 1.07 |
| D | 2006 | 80 | 52.3 | 1.47 | 3.60 | 46.03 | 49.63 | 9.34 | 37.77 | 47.11 | 0.95 |
| D | 2005 | 80 | 50 | 1.67 | 3.24 | 43.71 | 46.95 | 9.18 | 39.14 | 48.32 | 1.03 |
| D | 2004 | 80 | 68.5 | 1.00 | 4.88 | 42.99 | 47.87 | 12.52 | 34.88 | 47.40 | 0.99 |
| D | 2001 | 80 | 60.4 | 0.83 | 3.97 | 42.18 | 46.15 | 11.18 | 37.53 | 48.71 | 1.06 |
| D | 2007 | 120 | 2.9 | 2.96 | 0.94 | 37.33 | 38.27 | 1.79 | 22.62 | 24.41 | 0.64 |
| D | 2007 | 80 | 75.1 | 1.07 | 4.93 | 37.32 | 42.25 | 13.78 | 32.98 | 46.76 | 1.11 |
| D | 2009 | 80 | 49 | 1.40 | 2.68 | 36.95 | 39.62 | 8.08 | 35.25 | 43.33 | 1.09 |
| D | 2009 | 80 | 72.5 | 0.86 | 4.27 | 34.35 | 38.62 | 12.35 | 31.38 | 43.72 | 1.13 |
| D | 2006 | 80 | 70.3 | 0.95 | 3.65 | 30.88 | 34.54 | 11.48 | 30.69 | 42.17 | 1.22 |
| D | 2007 | 60 | 54.8 | 1.15 | 2.41 | 29.12 | 31.53 | 6.93 | 26.47 | 33.41 | 1.06 |
| A | 2003 | 71 | 8.9 | 1.21 | 0.63 | 21.90 | 22.53 | 1.62 | 17.82 | 19.44 | 0.86 |
| A | 2003 | 62 | 26 | 0.72 | 0.73 | 17.10 | 17.83 | 1.57 | 11.63 | 13.20 | 0.74 |
| A | 2003 | 55 | 26.2 | 0.78 | 0.62 | 14.56 | 15.18 | 1.85 | 13.69 | 15.54 | 1.02 |
| A | 2003 | 62 | 37 | 0.66 | 0.55 | 10.10 | 10.65 | 1.60 | 9.21 | 10.81 | 1.01 |
| C | 2006 | 65 | 70.08 | 3.42 | 4.05 | 34.41 | 38.46 | 8.07 | 21.68 | 29.75 | 0.77 |
| C | 2006 | 46 | 96.56 | 1.17 | 2.17 | 10.03 | 12.20 | 6.31 | 9.22 | 15.53 | 1.27 |
| B | 2003 | 20 | 38 |  | 0.35 | 6.27 | 6.62 | 1.38 | 7.77 | 9.15 | 1.38 |
| B | 2003 | 33 | 35 |  | 0.27 | 5.09 | 5.36 | 0.78 | 4.68 | 5.46 | 1.02 |
| A | 1990 | 137 | 1 |  | 0.26 | 10.77 | 11.03 | 0.22 | 2.85 | 3.06 | 0.28 |
| A | 1990 | 660 | 1 |  | 0.25 | 10.62 | 10.87 | 0.64 | 8.48 | 9.12 | 0.84 |
| A | 1990 | 650 | 1 |  | 0.22 | 9.17 | 9.39 | 0.83 | 10.96 | 11.79 | 1.26 |
| A | 1990 | 649 | 5 |  | 0.11 | 4.31 | 4.42 | 0.43 | 5.18 | 5.62 | 1.27 |
| A | 1990 | 470 | 4 |  | 0.18 | 7.08 | 7.26 | 0.30 | 3.71 | 4.02 | 0.55 |
| A | 1990 | 340 | 0 |  | 0.17 | 7.45 | 7.63 | 0.18 | 2.42 | 2.60 | 0.34 |
| A | 1990 | 650 | 3 | 0.52 | 0.15 | 6.16 | 6.31 | 0.34 | 4.24 | 4.58 | 0.72 |
| A | 1990 | 660 | 3 | 0.20 | 0.03 | 1.01 | 1.04 | 0.08 | 0.98 | 1.06 | 1.02 |
| A | 1990 | 560 | 5 | 0.42 | 0.10 | 3.75 | 3.85 | 0.18 | 2.11 | 2.29 | 0.59 |
| A | 1990 | 537 | 3 | 0.37 | 0.13 | 5.34 | 5.48 | 0.21 | 2.61 | 2.81 | 0.51 |
| A | 1990 | 366 | 2 | 0.46 | 0.23 | 9.30 | 9.52 | 0.46 | 5.98 | 6.45 | 0.68 |
| A | 1990 | 329 | 2 |  | 0.05 | 2.16 | 2.22 | 0.11 | 1.37 | 1.48 | 0.67 |
| A | 1990 | 202 | 1 |  | 0.23 | 9.42 | 9.64 | 0.28 | 3.69 | 3.97 | 0.41 |
| A | 1990 | 290 | 1 |  | 0.19 | 8.00 | 8.19 | 0.27 | 3.53 | 3.80 | 0.46 |
| A | 1990 | 256 | 1 |  | 0.04 | 1.86 | 1.91 | 0.07 | 0.87 | 0.94 | 0.49 |
| A | 1990 | 185 | 10 |  | 0.30 | 10.17 | 10.48 | 0.56 | 6.05 | 6.62 | 0.63 |
| A | 1990 | 185 | 1 |  | 0.22 | 8.97 | 9.18 | 0.60 | 7.86 | 8.46 | 0.92 |
| A | 1990 | 512 | 1 |  | 0.06 | 2.69 | 2.76 | 0.16 | 2.14 | 2.30 | 0.83 |
| A | 1990 | 478 | 1 |  | 0.13 | 5.46 | 5.59 | 0.21 | 2.77 | 2.98 | 0.53 |
| A | 1990 | 290 | 2 |  | 0.36 | 14.82 | 15.18 | 0.70 | 9.02 | 9.72 | 0.64 |
| A | 1989 | 318 | 5 |  | 0.55 | 20.88 | 21.42 | 0.82 | 9.87 | 10.69 | 0.50 |
| A | 1989 | 378 | 50 |  | 1.07 | 14.37 | 15.43 | 2.65 | 11.32 | 13.97 | 0.91 |
| A | 1989 | 325 | 1 |  | 0.20 | 8.41 | 8.62 | 0.18 | 2.41 | 2.59 | 0.30 |
| A | 1989 | 221 | 2 |  | 0.10 | 4.09 | 4.19 | 0.12 | 1.58 | 1.70 | 0.41 |
| A | 1989 | 221 | 38 |  | 1.71 | 30.45 | 32.16 | 3.45 | 19.38 | 22.82 | 0.71 |
| A | 1989 | 220 | 2 |  | 0.10 | 4.17 | 4.27 | 0.16 | 2.01 | 2.17 | 0.51 |
| A | 1989 | 348 | 9 |  | 0.08 | 2.80 | 2.88 | 0.30 | 3.31 | 3.61 | 1.25 |
| A | 1989 | 482 | 5 |  | 0.19 | 7.38 | 7.57 | 0.29 | 3.48 | 3.77 | 0.50 |
| A | 1989 | 580 | 1 |  | 0.05 | 2.14 | 2.19 | 0.09 | 1.13 | 1.21 | 0.55 |
| A | 1989 | 635 | 10 |  | 0.02 | 0.59 | 0.60 | 0.03 | 0.34 | 0.37 | 0.62 |
| A | 1989 | 588 | 1 |  | 0.02 | 0.76 | 0.78 | 0.04 | 0.57 | 0.61 | 0.78 |
| A | 1989 | 328 | 10 |  | 0.30 | 10.31 | 10.62 | 0.38 | 4.11 | 4.50 | 0.42 |
| A | 1989 | 388 | 10 |  | 0.07 | 2.28 | 2.34 | 0.17 | 1.78 | 1.95 | 0.83 |
| C | 2000 | 80 | 59.35 | 1.08 | 4.56 | 49.56 | 54.12 | 8.65 | 29.75 | 38.40 | 0.71 |
| C | 2000 | 75 | 56.94 | 1.12 | 3.19 | 36.72 | 39.91 | 8.53 | 31.00 | 39.52 | 0.99 |
| C | 2000 | 77 | 62.28 | 1.16 | 3.57 | 36.33 | 39.91 | 9.47 | 30.44 | 39.90 | 1.00 |
| C | 2000 | 70 | 53.38 | 1.16 | 1.89 | 23.53 | 25.42 | 5.07 | 20.02 | 25.09 | 0.99 |
| C | 2000 | 85 | 53.92 | 1.20 | 1.61 | 19.90 | 21.52 | 4.78 | 18.65 | 23.43 | 1.09 |
| C | 2000 | 76 | 59.84 | 1.13 | 1.81 | 19.48 | 21.29 | 5.36 | 18.23 | 23.58 | 1.11 |
| C | 2000 | 70 | 65.75 | 1.06 | 2.07 | 19.46 | 21.54 | 5.74 | 17.05 | 22.79 | 1.06 |
| B | 2001 | 34 | 78.2 |  | 4.78 | 33.72 | 38.50 | 15.09 | 33.63 | 48.72 | 1.27 |
| B | 2000 | 38 | 75.9 |  | 2.24 | 16.68 | 18.92 | 6.78 | 15.93 | 22.71 | 1.20 |
| B | 2001 | 38 | 70.7 |  | 1.81 | 15.18 | 17.00 | 6.08 | 16.11 | 22.20 | 1.31 |
| F | 2001 | 60 | 65.8 | 2.12 | 8.55 | 80.13 | 88.67 | 18.98 | 56.27 | 75.25 | 0.85 |
| F | 2002 | 54 | 62.5 | 1.22 | 7.43 | 75.13 | 82.56 | 16.85 | 53.90 | 70.74 | 0.86 |
| F | 2006 | 54 | 68.5 | 1.04 | 8.20 | 72.24 | 80.44 | 10.58 | 29.48 | 40.06 | 0.50 |
| F | 2006 | 58 | 66.5 | 1.89 | 7.26 | 67.01 | 74.27 | 11.72 | 34.18 | 45.90 | 0.62 |
| F | 2005 | 59 | 63.8 | 2.73 | 6.69 | 65.71 | 72.40 | 11.08 | 34.39 | 45.47 | 0.63 |
| F | 2002 | 60 | 63.8 | 2.12 | 6.64 | 65.14 | 71.78 | 16.15 | 50.14 | 66.28 | 0.92 |
| F | 2005 | 60 | 67.5 | 2.12 | 6.92 | 62.40 | 69.33 | 12.20 | 34.78 | 46.97 | 0.68 |
| F | 2006 | 59 | 65.7 | 1.98 | 6.52 | 61.29 | 67.81 | 9.25 | 27.47 | 36.72 | 0.54 |
| F | 2003 | 123 | 60.1 | 1.71 | 5.63 | 60.20 | 65.83 | 8.65 | 29.23 | 37.88 | 0.58 |
| F | 2000 | 123 | 65.9 | 1.39 | 6.41 | 60.00 | 66.42 | 3.96 | 53.49 | 57.45 | 0.86 |
| F | 2004 | 59 | 65.1 | 1.92 | 6.23 | 59.37 | 65.60 | 7.74 | 23.31 | 31.05 | 0.47 |
| F | 2004 | 58 | 65.2 | 1.76 | 6.21 | 59.04 | 65.25 | 10.31 | 30.99 | 41.31 | 0.63 |
| F | 2001 | 58 | 57 | 2.36 | 5.12 | 58.76 | 63.88 | 14.59 | 52.98 | 67.58 | 1.06 |
| F | 2000 | 57 | 80 | 0.50 | 8.64 | 58.42 | 67.06 | 22.43 | 47.96 | 70.40 | 1.05 |
| F | 2001 | 57 | 61.7 | 0.50 | 5.52 | 56.93 | 62.45 | 7.63 | 24.87 | 32.51 | 0.52 |
| F | 2002 | 59 | 69.5 | 0.50 | 6.38 | 54.96 | 61.35 | 10.06 | 27.38 | 37.44 | 0.61 |
| F | 2003 | 60 | 66.1 | 2.12 | 5.66 | 52.71 | 58.37 | 14.34 | 42.23 | 56.57 | 0.97 |
| F | 2005 | 54 | 68.5 | 1.15 | 5.88 | 51.79 | 57.67 | 13.15 | 36.64 | 49.80 | 0.86 |
| F | 2003 | 58 | 62.1 | 2.11 | 4.39 | 44.82 | 49.21 | 11.22 | 36.21 | 47.43 | 0.96 |
| F | 2005 | 58 | 62 | 2.27 | 4.33 | 44.35 | 48.68 | 11.63 | 37.64 | 49.27 | 1.01 |
| F | 2006 | 60 | 58.2 | 2.12 | 3.85 | 42.95 | 46.80 | 8.91 | 31.47 | 40.38 | 0.86 |
| F | 2004 | 54 | 66.63 | 0.85 | 4.67 | 42.92 | 47.58 | 9.51 | 27.67 | 37.18 | 0.78 |
| F | 2002 | 123 | 82.4 | 1.59 | 6.66 | 42.63 | 49.30 | 14.79 | 29.92 | 44.71 | 0.91 |
| F | 2003 | 54 | 62 | 1.27 | 4.10 | 41.97 | 46.07 | 11.06 | 35.79 | 46.85 | 1.02 |
| F | 2002 | 59 | 58.5 | 5.98 | 3.70 | 41.04 | 44.74 | 13.51 | 47.38 | 60.89 | 1.36 |
| F | 2002 | 58 | 59.2 | 1.41 | 3.66 | 39.97 | 43.64 | 14.60 | 50.38 | 64.97 | 1.49 |
| F | 2000 | 59 | 57.7 | 1.71 | 3.50 | 39.55 | 43.05 | 13.39 | 47.83 | 61.22 | 1.42 |
| F | 2000 | 61 | 70.7 | 0.50 | 4.69 | 39.26 | 43.95 | 18.15 | 48.08 | 66.23 | 1.51 |
| F | 2004 | 60 | 65.6 | 2.12 | 3.95 | 37.19 | 41.14 | 12.91 | 38.46 | 51.37 | 1.25 |
| F | 2000 | 60 | 56.4667 | 2.12 | 3.20 | 37.17 | 40.37 | 18.42 | 67.72 | 86.15 | 2.13 |
| F | 2005 | 123 | 67.3 | 1.56 | 4.03 | 36.46 | 40.49 | 9.50 | 27.22 | 36.73 | 0.91 |
| F | 2000 | 58 | 58.7 | 1.30 | 3.25 | 35.93 | 39.19 | 13.57 | 47.38 | 60.95 | 1.56 |
| F | 2001 | 59 | 72.8 | 0.50 | 4.48 | 35.79 | 40.27 | 11.83 | 29.85 | 41.68 | 1.04 |
| F | 2001 | 123 | 65.6 | 1.17 | 3.78 | 35.58 | 39.36 | 18.92 | 56.35 | 75.27 | 1.91 |
| F | 2006 | 123 | 68.5 | 1.57 | 3.90 | 34.39 | 38.29 | 12.54 | 34.93 | 47.47 | 1.24 |
| F | 2000 | 59 | 75.8 | 0.50 | 4.57 | 34.03 | 38.59 | 19.61 | 46.19 | 65.80 | 1.70 |
| F | 2003 | 59 | 62.6 | 1.96 | 3.37 | 33.99 | 37.35 | 10.73 | 34.25 | 44.99 | 1.20 |
| F | 2001 | 61 | 73.2 | 0.50 | 4.29 | 33.89 | 38.18 | 11.22 | 28.05 | 39.27 | 1.03 |
| F | 2001 | 54 | 59.5 | 1.07 | 3.05 | 33.09 | 36.14 | 18.67 | 64.00 | 82.67 | 2.29 |
| F | 2004 | 123 | 73.4 | 2.01 | 4.17 | 32.85 | 37.03 | 12.42 | 30.90 | 43.32 | 1.17 |
| F | 2003 | 59 | 72.3 | 0.50 | 3.92 | 31.64 | 35.55 | 9.87 | 25.20 | 35.08 | 0.99 |
| F | 2002 | 61 | 61.7 | 0.50 | 2.92 | 30.12 | 33.04 | 9.93 | 32.34 | 42.27 | 1.28 |
| F | 2000 | 54 | 58.7 | 1.10 | 2.71 | 29.95 | 32.67 | 11.72 | 40.91 | 52.63 | 1.61 |
| F | 2006 | 57 | 71.5 | 0.50 | 3.61 | 29.66 | 33.27 | 7.12 | 18.51 | 25.63 | 0.77 |
| F | 2004 | 59 | 75 | 0.50 | 3.79 | 28.77 | 32.56 | 7.27 | 17.44 | 24.72 | 0.76 |
| F | 2002 | 57 | 71.4 | 0.50 | 3.43 | 28.24 | 31.66 | 9.38 | 24.46 | 33.84 | 1.07 |
| F | 2005 | 57 | 66.1 | 0.50 | 2.78 | 25.89 | 28.68 | 7.98 | 23.51 | 31.49 | 1.10 |
| F | 2001 | 59 | 57.5 | 1.68 | 2.27 | 25.76 | 28.02 | 14.95 | 53.67 | 68.63 | 2.45 |
| F | 2005 | 61 | 54.3 | 0.50 | 2.04 | 24.98 | 27.02 | 4.52 | 17.48 | 22.00 | 0.81 |
| F | 2003 | 61 | 58.1 | 0.50 | 2.18 | 24.35 | 26.53 | 5.99 | 21.22 | 27.21 | 1.03 |
| F | 2006 | 61 | 77.285 | 0.52 | 3.34 | 24.02 | 27.36 | 9.58 | 21.81 | 31.39 | 1.15 |
| F | 2004 | 57 | 71.9 | 0.53 | 2.90 | 23.59 | 26.49 | 9.89 | 25.48 | 35.38 | 1.34 |
| F | 2005 | 61 | 59.6 | 0.51 | 2.14 | 23.14 | 25.28 | 6.33 | 21.63 | 27.96 | 1.11 |
| F | 2005 | 61 | 68.7 | 0.50 | 2.62 | 22.97 | 25.58 | 5.35 | 14.83 | 20.18 | 0.79 |
| F | 2006 | 61 | 59.6 | 0.50 | 2.07 | 22.43 | 24.50 | 5.34 | 18.26 | 23.60 | 0.96 |
| F | 2003 | 57 | 71.4 | 0.50 | 2.54 | 20.96 | 23.50 | 7.43 | 19.35 | 26.78 | 1.14 |
| F | 2005 | 59 | 51.5 | 0.52 | 1.57 | 20.41 | 21.98 | 5.87 | 24.19 | 30.06 | 1.37 |
| F | 2006 | 61 | 73 | 0.50 | 2.28 | 18.14 | 20.43 | 9.17 | 23.03 | 32.19 | 1.58 |
| F | 2006 | 61 | 48.3 | 0.50 | 1.16 | 16.21 | 17.36 | 4.58 | 20.32 | 24.90 | 1.43 |
| F | 2004 | 61 | 58.5 | 0.50 | 1.43 | 15.86 | 17.29 | 4.31 | 15.12 | 19.43 | 1.12 |
| F | 2003 | 55 | 73.9 | 0.79 | 3.12 | 24.24 | 27.36 | 9.54 | 23.46 | 33.00 | 1.21 |
| F | 2003 | 55 | 91.5 | 0.88 | 3.06 | 15.86 | 18.92 | 10.38 | 17.03 | 27.41 | 1.45 |
| A | 2001 | 9 | 26.4 | 3.64 | 1.39 | 32.37 | 33.76 | 4.14 | 30.41 | 34.56 | 1.02 |
| A | 2001 | 8 | 69.5 | 0.81 | 2.64 | 22.71 | 25.35 | 8.57 | 23.32 | 31.89 | 1.26 |
| A | 2001 | 19 | 62.5 | 0.86 | 1.44 | 14.58 | 16.02 | 5.29 | 16.91 | 22.20 | 1.39 |
| D | 1988 | 6 | 99.6 | 0.52 | 5.23 | 22.50 | 27.72 | 21.74 | 29.60 | 51.34 | 1.85 |
| D | 1988 | 2 | 93 | 0.51 | 4.37 | 21.90 | 26.27 | 16.93 | 26.83 | 43.76 | 1.67 |
| D | 1988 | 38 | 67 | 1.01 | 2.06 | 18.82 | 20.88 | 3.51 | 10.12 | 13.64 | 0.65 |
| D | 1988 | 11 | 99 | 1.12 | 4.04 | 17.65 | 21.70 | 17.36 | 23.96 | 41.31 | 1.90 |
| D | 1988 | 37 | 60 | 0.95 | 1.56 | 16.67 | 18.22 | 3.23 | 10.93 | 14.16 | 0.78 |
| D | 1988 | 1 | 99.9 | 0.41 | 2.81 | 12.03 | 14.84 | 13.63 | 18.43 | 32.06 | 2.16 |
| D | 1988 | 27 | 61 | 0.93 | 1.14 | 11.90 | 13.03 | 3.02 | 10.00 | 13.02 | 1.00 |
| D | 1988 | 6 | 99.4 | 0.35 | 2.50 | 10.80 | 13.30 | 10.39 | 14.21 | 24.60 | 1.85 |
| D | 1988 | 1 | 99.7 | 0.32 | 2.47 | 10.60 | 13.07 | 9.62 | 13.07 | 22.69 | 1.74 |
| D | 1988 | 7 | 99.2 | 0.41 | 1.89 | 8.22 | 10.11 | 8.21 | 11.28 | 19.50 | 1.93 |
| D | 1988 | 5 | 99.8 | 0.38 | 1.80 | 7.71 | 9.51 | 7.12 | 9.65 | 16.76 | 1.76 |
| D | 1988 | 5 | 97 | 0.42 | 0.96 | 4.37 | 5.32 | 4.53 | 6.55 | 11.09 | 2.08 |
| D | 1988 | 7 | 99.6 | 0.30 | 0.89 | 3.85 | 4.74 | 4.69 | 6.38 | 11.07 | 2.33 |
| D | 1988 | 9 | 99.8 | 0.26 | 0.45 | 1.95 | 2.40 | 1.66 | 2.24 | 3.90 | 1.63 |
| D | 1988 | 2 | 99.9 | 0.26 | 0.36 | 1.55 | 1.91 | 1.43 | 1.94 | 3.37 | 1.77 |
| C | 2002 | 10 | 61.4 |  | 1.57 | 16.24 | 17.81 | 4.72 | 15.49 | 20.21 | 1.13 |
| C | 2002 | 10 | 60.9 |  | 1.50 | 15.79 | 17.29 | 6.10 | 20.25 | 26.35 | 1.52 |
| C | 2002 | 10 | 61.9 |  | 1.54 | 15.74 | 17.28 | 4.56 | 14.79 | 19.35 | 1.12 |
| C | 2002 | 10 | 59.6 |  | 1.45 | 15.69 | 17.14 | 4.37 | 14.94 | 19.31 | 1.13 |
| C | 2002 | 10 | 62.8 |  | 1.43 | 14.40 | 15.84 | 5.35 | 16.99 | 22.34 | 1.41 |
| C | 2002 | 10 | 59.4 |  | 1.26 | 13.73 | 15.00 | 4.11 | 14.12 | 18.23 | 1.22 |
| C | 2002 | 10 | 60.3 |  | 1.27 | 13.49 | 14.75 | 3.56 | 11.97 | 15.52 | 1.05 |
| C | 2002 | 10 | 62.8 |  | 1.26 | 12.65 | 13.91 | 4.76 | 15.12 | 19.89 | 1.43 |
| C | 2002 | 10 | 62.9 |  | 1.23 | 12.33 | 13.56 | 3.73 | 11.82 | 15.54 | 1.15 |
| C | 2002 | 10 | 62.9 |  | 1.23 | 12.33 | 13.56 | 3.73 | 11.82 | 15.54 | 1.15 |
| C | 2002 | 10 | 63.1 |  | 1.07 | 10.70 | 11.77 | 2.85 | 8.98 | 11.82 | 1.00 |
| C | 2002 | 10 | 56.2 |  | 0.81 | 9.47 | 10.28 | 3.09 | 11.44 | 14.54 | 1.41 |
| C | 2002 | 10 | 57.2 |  | 0.81 | 9.22 | 10.03 | 3.74 | 13.51 | 17.25 | 1.72 |
| C | 2002 | 10 | 60.5 |  | 0.84 | 8.94 | 9.78 | 2.05 | 6.87 | 8.92 | 0.91 |
| C | 2002 | 10 | 53.2 |  | 0.65 | 8.10 | 8.75 | 2.72 | 10.78 | 13.50 | 1.54 |
| C | 2002 | 10 | 65.2 |  | 0.80 | 7.64 | 8.45 | 4.07 | 12.22 | 16.29 | 1.93 |
| C | 2002 | 10 | 58.39 |  | 0.69 | 7.62 | 8.30 | 3.05 | 10.74 | 13.79 | 1.66 |
| C | 2002 | 10 | 58.39 |  | 0.69 | 7.62 | 8.30 | 3.05 | 10.74 | 13.79 | 1.66 |
| C | 2002 | 10 | 61 |  | 0.25 | 2.57 | 2.82 | 0.98 | 3.25 | 4.23 | 1.50 |
| C | 2002 | 10 | 56.6 |  | 0.16 | 1.83 | 1.99 | 0.44 | 1.61 | 2.05 | 1.03 |
| D | 2006 | 186 | 20 | 6.59 | 4.80 | 129.19 | 133.99 | 3.77 | 32.10 | 35.87 | 0.27 |
| E | 2003 | 136 | 1.4 | 0.64 | 1.72 | 71.21 | 72.94 | 2.27 | 29.67 | 31.94 | 0.44 |
| D | 2004 | 240 | 10 | 0.69 | 1.66 | 56.27 | 57.93 | 1.70 | 18.23 | 19.93 | 0.34 |
| D | 2006 | 366 | 5 | 1.88 | 1.38 | 52.54 | 53.92 | 1.74 | 20.86 | 22.60 | 0.42 |
| E | 2003 | 85 | 1.2 | 0.67 | 0.42 | 17.38 | 17.80 | 1.04 | 13.68 | 14.72 | 0.83 |
| B | 2007 | 309 | 4 | 0.27 | 0.44 | 17.09 | 17.53 | 0.46 | 5.68 | 6.14 | 0.35 |
| B | 2007 | 365 | 3 | 0.58 | 0.33 | 13.21 | 13.54 | 0.52 | 6.59 | 7.11 | 0.53 |
| B | 2004 | 170 | 40 | 0.40 | 0.41 | 6.90 | 7.30 | 0.43 | 2.33 | 2.76 | 0.38 |
