# Supplementary material for: Towards Predicting Basin-Wide Invertebrate Organic Biomass and Production in Marine Sediments from a Coastal Sea
Source: PLoS One. 2012 Jul 6;7(7):e40295. doi: 10.1371/journal.pone.0040295 (PMC3391270; doi:10.1371/journal.pone.0040295)
Supplement: Reference List S1 — (DOC) [file pone.0040295.s007.doc]

# Supporting Information References S1

S1 Burd B, Moore D, Brinkhurst RO (1987) Distribution and Abundance of Macrobenthic Fauna from Boundary and Mud Bays near the British Columbia/U.S. Border. Canadian Technical Report of Hydrography and Ocean Sciences 84: 1-34.

S2 Burd BJ, Glaholt R (2000a) Reconnaissance Level Baseline Survey of Benthic Infaunal communities at Ecological Reserve 67 and Adjacent Satellite Channel, June 4, 2000. Georgia Strait Crossing Pipeline Ltd. 14pp + appendices.

S3 Burd BJ, Brinkhurst RO (1992) Benthic Infaunal Surveys of British Columbia Fjords, 1988 to 1990. Canadian Data Report of Hydrography and Ocean Sciences 114. 37 p.

S4 Lynch BR, BJ Burd J Clarke, P Howland, T Tomliens, K Pillay, I Dyck (2011) Iona Deep-Sea Outfall Receiving Environment Monitoring Program, 2010 Sediment Effects Survey. Report Prepared for Metro Vancouver, Burnaby, BC by Worley Parsons Canada Services Ltd., Victoria, BC. 225 p. + Appendices.

S5 Burd BJ, Glaholt R (2000b) Survey of benthic infauna at the Manley Creek landfall site at Boatswain Bank, British Columbia. Ecostat Research ltd and Tera Environmental Consultants (Alta) Ltd. Report to the Georgia Strait Crossing (GSX) pipeline project.

S6 SeaEcology, Pacific Marine Life Surveys Inc., Tera Environmental Consutants (Alta.) Ltd., (2001) Biological Survey and Assessment of Benthic Habitats for a Proposed Manley Creek Pipeline Landfall at Boatswain Bank, British Columbia-July 2000. Prepared for Georgia Strait Crossing Pipeline Ltd. 47 p. + appendices.

S7 G3 Consulting (2003) Britannia Beach Subtidal Sampling Programs: Final Report. Prepared for Environment Canada. 62 p.

S8 Barnes PAG (2006) Shellfish Culture and Particulate Matter Production and Cycling: A Literature Review. B.C. Aquaculture Research and Development Committee. Project AE02.03-02.01. 101 p.

S9 McPherson CA, Chapman PM, McKinnon SJ, Burd BJ, Fanning ML, Olson J, Pillay K, Ross-Easton H (2012) Lions Gate Outfall Receiving Environment Monitoring Program, 2010 Sediment Effects Survey. Draft report prepared for Metro Vancouver, Burnaby, BC by Golder Associates Ltd., Burnaby, BC.

S10 Wright C, Taekeama B, Burd B, McGreer, E (2007a) Salmon Aquaculture Environmenal Monitoring Data Report. Results of Sampling Program for Year 2000. A Report Prepared for the Ministry of the Environment, Province of British Columbia, Nanaimo, B.C.

S11 Wright C, Taekeama B, Burd B, McGreer, ER (2007c) Salmon Aquaculture Environmenal Monitoring Data Report. Results of Sampling Program for Year 2002. A Report Prepared for the Ministry of the Environment, Province of British Columbia, Nanaimo, B.C.

S12 Wright C, Taekeama B, Burd B, McGreer ER (2007d) Salmon Aquaculture Environmenal Monitoring Data Report. Results of Sampling Program for Year 2003. A Report Prepared for the Ministry of the Environment, Province of British Columbia, Nanaimo, B.C.

S13 Wright C, Taekeama B, Burd B, McGreer ER (2007e) Salmon Aquaculture Environmenal Monitoring Data Report. Results of Sampling Program for Year 2004. A Report Prepared for the Ministry of the Environment, Province of British Columbia, Nanaimo, B.C.

S14 Wright C, Taekema B, Burd B, Dalby Jr J, McGreer ER (2007b) Salmon Aquaculture Environmenal Monitoring Data Report. Results of Sampling Program for Year 2001. A Report Prepared for the Ministry of the Environment, Province of British Columbia, Nanaimo, B.C.

S15 Wright CA, Johannessen SC, Macdonald RW, Burd BJ, Hill PR, van Roodselaar A, Bertold S (2008) The Strait of Georgia Ambient Monitoring Program, Phase I 2002-2007: Sediment and Benthos. Canadian Data Report of Fisheries and Aquatic Sciences 1208. 112p.

S16 McPherson CA, Chapman MK, Fanning ML, Olson J, Chen, F (2004) Georgia Strait Ambient Monitoring Program - Data Report. Prepared for the Greater Vancouver Regional District by EVS Consultants Ltd, Burnaby, B.C. 28 p. + appendices.

S17 Paine Ledge and Associates (2004) Trend analysis of Macauley Point Invertebrate Community Data 1994-2002. Prepared for the Capital Regional District, Environmental Services Department., Victoria, B.C.

S18 Glaholt R, Burd B, Haight, R (2002) Preliminary Report on the Environmental Effects of a Marine Pipeline on Nearby Soft Bottom Benthic Infaunal Communities, Bazan Bay, British Columbia. Prepared for Georgia Strait Crossing Pipeline Limited, Prepared by Tera Environmental Services, Ecostat Research Ltd., and Vacilador Productions Ltd., 21 p. + appendices.

S19 Associated Engineering, Lorax Environmental (2005) Greater Nanaimo Pollution Control Centre: A summary of receiving environment monitoring studies and a proposed receiving environment monitoring program (Final Report for Regional District of Nanaimo, BC. June 6, 2005).

S20 Yunker MB, Macdonald RW, Goyette D, Paton DW, Fowler BR, Sullivan D, Boyd J (1999) Natural and anthropogenic inputs of hydrocarbons to the Strait of Georgia. Science of the Total Environment 225: 181-209.

S21 Johannessen SC, O'Brien MC, Denman KL, Macdonald RW (2005a) Seasonal and spatial variations in the source and transport of sinking particles in the Strait of Georgia, British Columbia, Canada. Marine Geology: 216, 59-77.

S22 Johannessen SC, Macdonald RW, Eek, KM (2005b) Historical trends in mercury sedimentation and mixing in the Strait of Georgia, Canada. Environmental Science & Technology 39: 4361-4368.

S23 Macdonald RW, Cretney WJ, Crewe N, Paton D (1992) A history of octachlorodibenzo-p-dioxin, 2,3,7,8-tetrachlorodibenzofuran and 3,3', 4-4'-tetrachlorobiphenyl contamination in Howe Sound, British Columbia. Environmental Science & Technology 26: 1544-1550.

S24 Shang DY, Macdonald RW, Ikonomu MG (1999) Persistence of nonylphenol and ethyoxylate surfactants and their principle primary degradation products in sediments from near a municipal outfall in the Strait of Georgia, British Columbia, Canada. Environmental Science & Technology 33: 1366-1372.

S25 Boyd J, Macdonald R, Paton DW, Hutton K, Baumann J, Bertold S, Moore B (1997) Burrard Inlet Sediment Core Contaminant Profiles. A Report Prepared for the Burrard Inlet Environmental Action Program, Vancouver, BC. 15 p. + appendices

S26 Picard K, Hill PR, Johannessen SC (2006) Sedimentation rates and surficial geology in the Canadian Forces Maritimes Experimental and Test Range exercise area Whiskey Golf, Strait of Georgia, British Columbia. In: Current Research no. 2006-A5, Geological Survey of Canada, Pacific Geoscience Centre, Sidney, BC. 9p.

S27 Dinn, P Johannessen, S, Macdonald, R, Lowe, C, Whiticar, M (In press). Effect of receiving environment on the transport and fate of polybrominated diphenyl ethers near two submarine outfalls, by Environmental Toxicology and Chemistry .

S28 Carpenter R, Bennett J, Peterson M (1981) 210Pb activities in and fluxes to sediments of the Washington continental slope and shelf. Geochimica et Cosmochimica Acta 45: 1155-1172.

S29 Timothy DA, Soon M, Calvert S (2003) Settling fluxes in Saanich and Jervis Inlets, British Columbia, Canada: sources and seasonal patterns. Progress in Oceanography 59: 31-73.

S30 Timothy DA (2004). Organic matter remineralisation andbiogenic silica dissolution in a deep fjord in British Columbia, Canada: a regression analysis of upper ocean sediment-trap fluxes. Deep-Sea Research I 51: 429-456.

S31 Galeron J, Sibuet M, Mahaut M-L, Dinet A (2000) Variation in structure and biomass of the benthic communities at three contrasting sites in the tropical Northeast Atlantic. Marine Ecology Progress Series 197: 121-137.

S32 Brey T (2001) Population dynamics in benthicinvertebrates. A virtual handbook. Alfred Wegener Institute for Polar and Marine Research, Germany. <http://www.awi-bremerhaven.de/Benthic/Ecosystem/FoodWeb/Handbook/main.html>.

S33 Steimle F, Terranova R (1985) Energy equivalents of amrine organisms from the continental shelf of the temperate northwest Atlantic. Journal of Northwest Atlantic Fisheries Science 6: 117-124.

S34 Frithsen JB, Rudnick DT, Doering PH (1986). The determination of fresh organic carbon weight from formaldehyde preserved macrofaunal samples. Hydrobiologia 133: 203-208.

S35 Ricciardi A, Bourget E (1998) Weight-to-weight conversion factors for marine benthic macroinvertebrates Marine Ecology Progress Series 163: 245-251.

S36 Clarke A (2008) Ecological stoichiometry in six species of Antarctic marine benthos. Marine Ecology Progress Series 369: 25-37.

S37 Lie U (1968) A quantitative study of benthic infauna in Puget Sound. Fiskeridirektoratet. Skrifter. Serie Havundersoekelser 14: 556p.

S38 Cauffope G, Heymans S (2005) Energy contents and conversion factors for sea lion?s prey. UBC Fisheries Centre Research Reports 13: 1.

S39 Rowe G (1983) Biomass and production in the deep-sea macrobenthos. In: Rowe G. (Ed.) The sea. Deep-Sea Biology Volume 8. Wiley, New York: 97-121.

S40 Rudnick DT, Elmgren R, Frithsen J (1985) Meiofaunal prominence and benthic seasonality in a coastal marine ecosystem. Oecologia 67: 157-168.

S41 Leuven RS, Brock TC, van Druten HA (1985) Effects of preservation on dry- and ash-free dry weight biomass of some common aquatic macro-invertebrates. Hydrobiologia 127: 151-159.

S42 Fielman KT, Targett N M (1995) Variation of 2,3,4=tribromopyrrole and its sodium sulfamate salt in the hemichordate *Saccoglossus kowalevskii* Marine Ecology Progress Series 116: 125-136.

S43 Wacasey J, Atkinson E (1987) Energy values of marine benthic invertebrates from the Canadian Arctic. Marine Ecology Progress Series 39: 243-250.
